# Unlocking the Secrets Behind Advanced Artificial Intelligence Language Models in Deidentifying Chinese-English Mixed Clinical Text: Development and Validation Study

**DOI:** 10.2196/48443

**Published:** 2024-01-25

**Authors:** You-Qian Lee, Ching-Tai Chen, Chien-Chang Chen, Chung-Hong Lee, Peitsz Chen, Chi-Shin Wu, Hong-Jie Dai

**Affiliations:** 1 Dialogue System Technical Department Intelligent Robot Asustek Computer Inc Taipei Taiwan; 2 Intelligent System Laboratory, Department of Electrical Engineering College of Electrical Engineering and Computer Science National Kaohsiung University of Science and Technology Kaohsiung Taiwan; 3 Department of Bioinformatics and Medical Engineering Asia University Taichung Taiwan; 4 Center for Precision Health Research Asia University Taichung Taiwan; 5 Electromagnetic Sensing Control and AI Computing System Laboratory, Department of Electrical Engineering College of Electrical Engineering and Computer Science National Kaohsiung University of Science and Technology Kaohsiung Taiwan; 6 Knowledge Discovery and Data Mining Lab, Department of Electrical Engineering College of Electrical Engineering and Computer Science National Kaohsiung University of Science and Technology Kaohsiung Taiwan; 7 Department of Chemical Engineering Feng Chia University Taichung Taiwan; 8 National Center for Geriatrics and Welfare Research National Health Research Institutes Zhunan Taiwan; 9 National Institute of Cancer Research National Health Research Institutes Tainan Taiwan; 10 School of Post-Baccalaureate Medicine College of Medicine Kaohsiung Medical University Kaohsiung Taiwan; 11 Center for Big Data Research Kaohsiung Medical University Kaohsiung Taiwan

**Keywords:** code mixing, electronic health record, deidentification, pretrained language model, large language model, ChatGPT

## Abstract

**Background:**

The widespread use of electronic health records in the clinical and biomedical fields makes the removal of protected health information (PHI) essential to maintain privacy. However, a significant portion of information is recorded in unstructured textual forms, posing a challenge for deidentification. In multilingual countries, medical records could be written in a mixture of more than one language, referred to as code mixing. Most current clinical natural language processing techniques are designed for monolingual text, and there is a need to address the deidentification of code-mixed text.

**Objective:**

The aim of this study was to investigate the effectiveness and underlying mechanism of fine-tuned pretrained language models (PLMs) in identifying PHI in the code-mixed context. Additionally, we aimed to evaluate the potential of prompting large language models (LLMs) for recognizing PHI in a zero-shot manner.

**Methods:**

We compiled the first clinical code-mixed deidentification data set consisting of text written in Chinese and English. We explored the effectiveness of fine-tuned PLMs for recognizing PHI in code-mixed content, with a focus on whether PLMs exploit naming regularity and mention coverage to achieve superior performance, by probing the developed models’ outputs to examine their decision-making process. Furthermore, we investigated the potential of prompt-based in-context learning of LLMs for recognizing PHI in code-mixed text.

**Results:**

The developed methods were evaluated on a code-mixed deidentification corpus of 1700 discharge summaries. We observed that different PHI types had preferences in their occurrences within the different types of language-mixed sentences, and PLMs could effectively recognize PHI by exploiting the learned name regularity. However, the models may exhibit suboptimal results when regularity is weak or mentions contain unknown words that the representations cannot generate well. We also found that the availability of code-mixed training instances is essential for the model’s performance. Furthermore, the LLM-based deidentification method was a feasible and appealing approach that can be controlled and enhanced through natural language prompts.

**Conclusions:**

The study contributes to understanding the underlying mechanism of PLMs in addressing the deidentification process in the code-mixed context and highlights the significance of incorporating code-mixed training instances into the model training phase. To support the advancement of research, we created a manipulated subset of the resynthesized data set available for research purposes. Based on the compiled data set, we found that the LLM-based deidentification method is a feasible approach, but carefully crafted prompts are essential to avoid unwanted output. However, the use of such methods in the hospital setting requires careful consideration of data security and privacy concerns. Further research could explore the augmentation of PLMs and LLMs with external knowledge to improve their strength in recognizing rare PHI.

## Introduction

### Background

In the clinical and biomedical fields, electronic health records (EHRs) have become valuable resources in recent years [1]. In order to protect the privacy of patients whose data are used for secondary purposes, regulations or laws have been established requiring the removal of protected health information (PHI) from records before they can be disseminated. In settings, such as research, obtaining explicit consent to access personal data may be impractical or impossible. As a result, the deidentification step becomes a critical data processing step to protect the privacy of individuals. However, the study by Cannon and Lucci [2] indicated that up to 65% of important clinical information is recorded in unstructured texts in medical reports written by medical personnel. Compared with structured data, which can be deidentified by encrypting private patient information fields, the deidentification of unstructured data is more challenging.

Because manual deidentification of large volumes of EHRs is time-consuming and error-prone, automated methods are needed for large-scale deidentification of unstructured clinical data. Compared with Asian countries, such as Japan and China, where the majority of medical records are written monolingually in their native languages, medical records in Taiwan are frequently written in a mixture of Chinese and English. The example sentence “前夫mk1300309 married > mk136從婆家搬出來 > mk139離婚” (got married to my ex-husband on 2141/03/09 > moved out from the ex-husband’s family’s home in 2147 > divorced in 2150) demonstrates how a physician wrote in English and suddenly switched to Chinese. The sentence also includes a transliteration (“mk”) of “民國,” which refers to the Republic of China calendar. This phenomenon is referred to as code alternation, which occurs when a bilingual speaker uses more than one language in a single utterance [3]. Code switching refers to code alternation at or above clause level, while code mixing refers to code alteration below clause level. Some examples of code-switched and code-mixed narratives in discharge summaries are shown in Multimedia Appendix 1. Throughout this work, we will use code mixing to refer to the above phenomenon.

In addition to our specific context, it is important to note that code mixing in clinical records is observed in various multilingual health care settings globally. For instance, Alqurashi [4] conducted research on the use of code-mixed language in private hospitals in Saudi Arabia, highlighting the prevalence of this practice in health care communication. Furthermore, Dowlagar and Mamidi [5] demonstrated that the use of code-mixed utterances, where native languages are blended with English, is a common and natural occurrence among doctors and patients. Beyond this, code mixing in clinical notes extends beyond English-dominant countries. For example, Keresztes [6] investigated how Hungarian physicians are influenced by the English language in their writing of cardiology discharge reports, while Karuthan [7] showed that nurses in Malaysia tend to use “Manglish,” a mix of Malayalam and English, when writing nursing documents.

This global prevalence of code mixing highlights the importance of addressing narratives written in this manner. Unfortunately, most existing clinical natural language processing (NLP) techniques are primarily designed for monolingual texts, mainly due to limited research resources. While our previous work [8] has highlighted the potential of pretrained language models (PLMs) in mitigating issues related to code mixing during deidentification, the precise mechanism at play remains incompletely understood. To bridge this knowledge gap, there is a pressing need for a code-mixed deidentification data set that can comprehensively evaluate the performance of state-of-the-art NLP models and unravel their underlying mechanisms.

### Goal of This Study

Previous studies [9,10] have demonstrated that state-of-the-art neural network models can effectively exploit strong name regularity and high mention coverage to achieve superior performance. Name regularity refers to the consistency and predictability of the structure and pronunciation of a named entity type within a particular language. For example, person names typically adhere to the “FirstName LastName” format. A high mention coverage denotes that a large proportion of mentions in the test set have been previously observed in the training set.

Our study aims to explore the impact of these properties on the performance of PLMs in recognizing PHI within code-mixed sentences. We hypothesize that the decision-making processes of the models are influenced by these factors, even when dealing with code-mixed text. However, they may underperform when faced with weak regularity, such as unseen mentions or out-of-vocabulary (OOV) words. Additionally, they may solely memorize popular mentions instead of learning generalization knowledge, which could limit their generality and performance. To investigate the decision-making process of these models and gain insights into their ability to recognize code-mixed PHI, we curated a novel code-mixed deidentification data set, trained and evaluated the performance of state-of-the-art PLMs on the unique data set, and employed methods to interpret their results to validate our hypothesis and gain a deeper understanding of how these models recognize code-mixed PHI.

In summary, this study has the following 3 major contributions:

1. Unique code-mixed deidentification data set: We significantly extended our original corpus compiled in our previous work [8] by incorporating an additional 900 discharge summaries. Furthermore, we created a manipulated subset of the resynthesized data set available for research purposes. Importantly, our work resulted in the creation of the very first clinical code-mixed data set that encompasses both traditional Chinese and English. This unique data set [11] represents a valuable resource for researchers, offering a fresh perspective on issues related to code mixing in the context of health care data and contributing to advancing research in code-mixed clinical text processing.
2. Enhancing the understanding of PLMs in code-mixed texts: The curated data set enables an in-depth exploration of the effectiveness and underlying mechanism of fine-tuned PLMs in recognizing code-mixed PHI. Through thorough analyses, we gained insights into the mechanism used by these models for this specific task.
3. Unprecedented exploration of large language models (LLMs) on code-mixed clinical text: The emergence of LLMs has raised a question about their potential application in the deidentification process. This study focused on the feasibility of employing prompt-based, zero-shot, in-context learning with LLMs to recognize PHI. More specifically, we leveraged ChatGPT to recognize PHI within code-mixed text that was previously challenging for the developed PLMs. To the best of our knowledge, this is the first attempt to use prompt-based in-context learning of LLMs for deidentification in code-mixed text.

### Related Work

#### Definition of PHI Types

The Health Insurance Portability and Accountability Act (HIPAA) is a federal law in the United States enacted in 1996 to protect the privacy and security of the PHI of individuals [12]. The Safe Harbor method under the HIPAA includes the removal of 18 specific identifiers from PHI, such as name, date of birth, and social security number. If all 18 identifiers are removed, the information is considered deidentified under the method, and the data can be reused for secondary purposes [13]. Given the sensitive nature of the clinical text used in this study, we expanded the list of PHI types to 20 fine-grained categories within 6 coarse-grained categories. The details are presented in Table 1.

**Table 1:** The coarse-grained and fine-grained categories of protected health information defined in the annotation guideline used in this study.

Coarse-grained PHI^a^	Fine-grained PHI
Date	Date
Age	Age
Name	Patient, person, doctor
Location	Named location, nationality, region, country, city, hospital, department, room, number, school, generic location, market
Profession	Profession
ID	ID number, medical record

^a^PHI: protected health information.

#### Publicly Available Deidentification or Code-Mixed Data Sets

A code-mixed named entity recognition competition was held in 2018 [14], in which twitter data with the mixture of English and Spanish, and Arabic and Egyptian were provided. The former consisted of 67,223 tweets, and the latter consisted of 12,334 tweets. To the best of our knowledge, there is only 1 publicly available Chinese-mixed corpus, which is a simple Chinese-English code-mixed data set for the task of emotion detection [15].

On the other hand, the MIMIC (Medical Information Mart for Intensive Care) II/III database [16] is one of the largest deidentified clinical databases on patients admitted to a medical center in the United States, which offers clinical text in English for researchers to access and use under a data access agreement. To facilitate automated deidentification tasks, large annotated corpora of unstructured text with PHI entities are required. However, the development of such corpora is complex and limited in number. To address this need, the Informatics for Integrating Biology and the Bedside (i2b2) has organized several clinical deidentified NLP competitions [17], such as the deidentified competition in 2014, which released a corpus of 1304 medical records of 296 patients with diabetes [18]. Another is the CEGS N-GRID deidentification corpus [19], containing 1000 psychiatric notes from the United States. Alla et al [20,21] compiled the first open available deidentification corpus from Australia, which contained 2100 pathology reports for the purpose of automatic deidentification. However, deidentification corpora in languages other than English are scarce. A Norwegian synthetic clinical corpus released by Bråthen et al [22] is an example, and it includes 477 sentences. To the best of our knowledge, there is currently no openly available code-mixed deidentification corpus.

#### Deidentification Methods and Approaches for Tackling Code-Mixing Challenges

Manual deidentification is costly, in terms of both finances and time. For example, deidentification of 50,000 patient visit records in the MIMIC-III data set can cost around US $500,000 and 5000 hours. To overcome these challenges, automated deidentification systems have been developed. Initially, rule-based systems using lexical dictionaries, regular expressions, and simple heuristics were introduced [23]. However, they have limitations in generalization across different data sets. Subsequently, machine learning–based approaches [23-25] were proposed, offering better generalization and prediction accuracy. Nonetheless, these methods still rely on handcrafted features. More recently, deep learning techniques have become mainstream, eliminating the need for manually crafted features [26,27]. Neural networks are advantageous because they can be initialized with PLMs acquired from extensive unlabeled data, resulting in faster optimization and superior performance. BERT (Bidirectional Encoder Representations from Transformers) pretrained on English corpora (EN-BERT) [28] is one such example of a monolingual transformer model pretrained on the BookCorpus [29] and English Wikipedia in a self-supervised fashion, which has achieved exceptional precision in various NLP tasks including the deidentification task [8,30].

On the other hand, the rise of code-mixed data sets has spurred the development of approaches designed to address the unique challenges posed by code-mixed content. For instance, in the work by Winata et al [31], a bidirectional long-short term memory model was used to leverage both character and word information, complemented by pretrained FastText embeddings [32], to improve the identification of OOV words in an English-Spanish mixed data set. Another approach by Trivedi et al [33] incorporated FastText word vectors [34] trained from Spanish Wikipedia pages and pretrained word embeddings sourced from a substantial collection of tweets [35] to tackle the English-Spanish code-mixing problem. They applied the singular value decomposition [36] method to align word embeddings across different languages, facilitating a unified vector space representation. Devlin et al [28] introduced pretrained models beyond monolingual EN-BERT, including BERT pretrained on simplified and traditional Chinese corpora (CH-BERT) and BERT pretrained on Wikipedia corpora from 104 languages (M-BERT). Researchers have been exploring the use of M-BERT to tackle the code-mixing problem owing to its ability to generalize cross-lingually through its multilingual representation [37]. For example, Tang et al [15] fine-tuned M-BERT on their English-simplified Chinese social media data set for multi-label sentiment analysis, and obtained an F-score of 0.69, which was 15% higher than that for the model without M-BERT. While limited research exists on using BERT-based models or other LLMs for code-mixing deidentification in clinical data sets, our previous work [38] suggested the potential benefits of incorporating M-BERT to disambiguate PHI categories in code-mixed sentences. In this study, we focused on comprehending how BERT-family PLMs handle Chinese-English mixed issues that arise in actual clinical text and assessed the feasibility of using state-of-the-art LLMs to recognize PHI from sampled clinical text.

## Methods

### Data Sources and Corpus Construction

We obtained discharge summaries sampled from the psychiatry section of a medical center, which were used to extract depressive symptoms with text mining approaches [39,40]. From these data, we compiled a code-mixed deidentification corpus of 1700 discharge summaries. Four annotators were enlisted to annotate the entire corpus, and they followed the same annotation procedure as previously described in our work [8]. To ensure the quality of the annotation, the annotation process began with the annotators individually annotating an identical set of 200 randomly sampled records, using the annotation guidelines provided. Subsequently, a meeting was organized to facilitate discussions among the annotators, addressing any issues or concerns that arose during the annotation process. This iterative process continued until the annotators achieved a strong level of agreement [41]. The interannotation agreement, as measured by κ, was found to be 0.85 after the above procedure. Once this agreement threshold was reached, the remaining unlabeled EHRs were evenly distributed among the 4 annotators for labeling. The training set comprised 1500 discharge summaries along with 297,621 sentences. The test set comprised 200 discharge summaries along with 60,632 sentences. Finally, the principle-based resynthesis method proposed in our previous work [38] was used to generate surrogates, and the entire corpus was rechecked by one of the senior annotators (PTC) to ensure a high level of data consistency and correctness.

### Ethics Approval

The study has been approved by the ethics committee for medical research of the National Institutes of Health (number: EC1090212-E).

### Implementation of Deidentification Methods

After preprocessing the collected data set, we approached the deidentification task by treating it as a named entity recognition problem in which the target entity types are PHI types defined in Table 1. To establish a baseline, we used a dictionary-based method [42,43] and developed 4 BERT-based models for comparison. The dictionary-based approach (DBA) relies on predefined dictionaries and a lookup method. In our implementation, we compiled a dictionary consisting of tokens for all PHI types collected from the training set. The most frequent tag associated with each token estimated on the training set was set as the token’s tag. For the BERT-based models, the recognition task was formulated as a sequential tagging problem by using the BILOU tagging schema. This schema categorizes the tokens as either the beginning (B), within (I), or last (L) of multi-word PHI, as well as identifies non-PHI (O) and single-word PHI (U) tokens. For more in-depth information about the preprocessing steps and the development of the DBA, please refer to Multimedia Appendix 2.

#### BERT-Based Approach

The BERT-family PLMs, including EN-BERT, CH-BERT, and M-BERT, were used in this study. In our implementation, an additional fully connected layer was added that takes the token embeddings from the top layer of the underling PLM as input. This layer is employed to predict the probabilities of the tags for each token. When the WordPiece tokenizer splits a token into multiple pieces, we take the representation of the first piece to represent the token [44]. Details of the procedure and hyperparameters used for training our BERT-based models are described in Multimedia Appendix 2.

**Figure 1:**
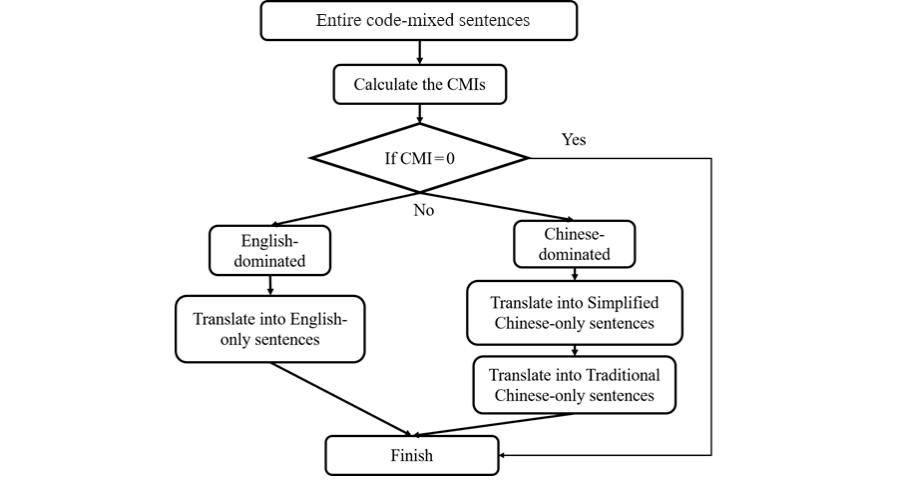
The procedure to generate the training and test sets for TM-BERT. CMI: code-mixing index; M-BERT: BERT pretrained on Wikipedia corpora from 104 languages; TM-BERT: M-BERT fine-tuned on the translated deidentification corpus.

In addition to fine-tuning the above BERT-based models on the original code-mixed corpus, we translated sentences in our English-Chinese corpus into either English or Chinese and then fine-tuned the M-BERT model on the translated deidentification corpus (TM-BERT) by determining the degree of code mixing present in a given sentence using the code-mixing index (CMI) [8,45]. The flowchart of the translation process for compiling the training set is shown in Figure 1.

We determined the degree of code mixing present in a given sentence *s* using the following formula proposed by Gambäck and Das [45]:



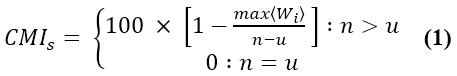



where *W_i_* is the number of tokens of the most frequent language in the sentence *s*, *n* is the total number of tokens, and *u* is the quantity of tokens in *s* that are independent of any particular language, such as numeric words and punctuation marks.

By definition, the CMI is always smaller than 0.5 for code mixing of 2 languages. A larger CMI (close to 0.5) indicates serious code mixing, while a smaller CMI (close to 0) suggests that 1 language dominates the majority of tokens. The CMI is set as zero if the entire sentence is either monolingual or composed of numeric words or punctuations. In cases where the CMI of a sentence is not zero, the number of tokens belonging to English and Chinese in the sentence is calculated. If the majority of tokens belong to English, the sentence is translated to English using a machine translation model [46]. Conversely, if Chinese tokens dominate, we opt for translation to traditional Chinese [47]. Subsequently, we proceeded with the identical fine-tuning process on the translated corpus. In the prediction phase, we employed identical procedures to transform the input sentences into monolingual sentences based on the dominating language and subsequently used TM-BERT to detect PHI.

#### ChatGPT-Based Deidentification Framework

**Figure 2:**
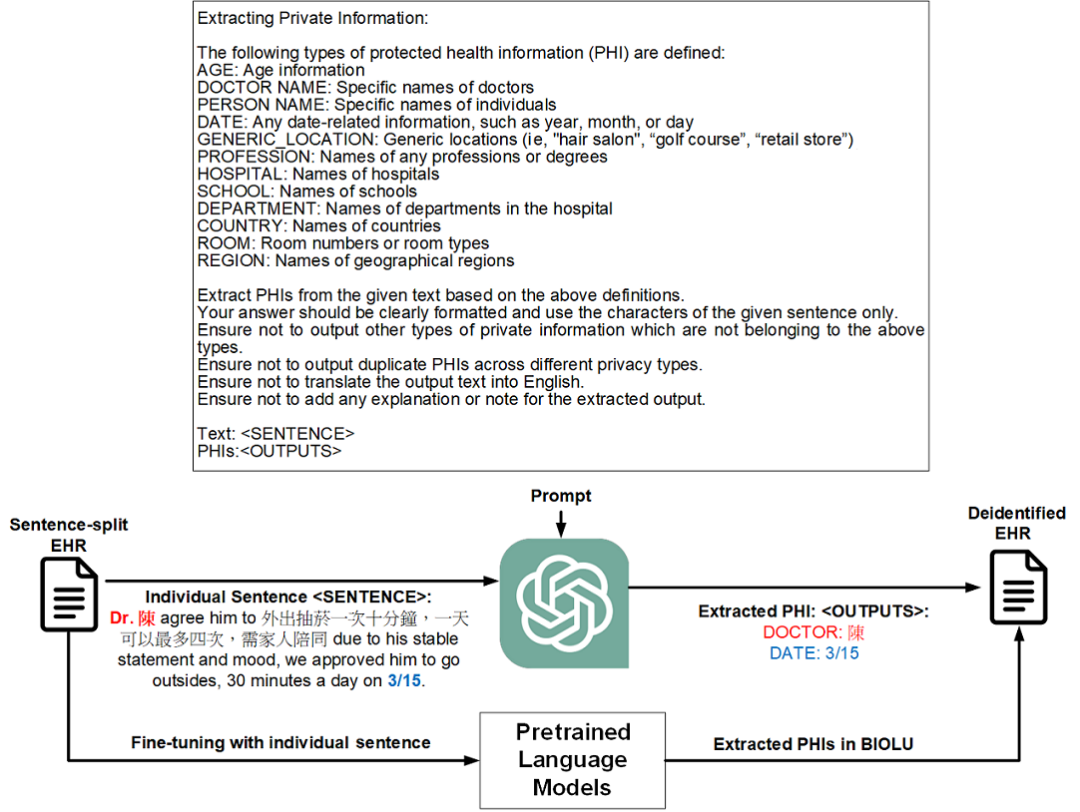
The developed framework for the recognition of protected health information mentioned in code-mixed text based on ChatGPT. EHR: electronic health record; PHI: protected health information.

Figure 2 presents our framework to use ChatGPT for the recognition of PHI, which is based on recent research suggesting that LLMs can be adapted to downstream tasks through the use of prompts [48]. A prompt is a set of instructions that can customize an LLM’s capabilities and affect its subsequent interactions and outputs. To optimize our prompt, we performed prompt engineering based on the PHI definitions. The objective was to ensure that the extracted candidate PHI text could be matched with the original input text represented by the “<SENTENCE>” placeholder. Following the prompt design procedure [49], we organized our prompt into 4 main parts:

1. Overall task instruction: This part provides general instructions for the task. We declared it as “Extracting Private Information.”
2. Sentence introduction: This part defines the placeholder for the input text (“<SENTENCE>”) and the PHI that we focus on.
3. Constraint: As illustrated in Figure 2, the text following the statement “Extract PHI from the given text based on the above definitions” forms the constraint part. The inclusion of this constraint is based on our observation that ChatGPT has the capability to directly extract various PHI types from text and sometimes translate extracted Chinese text into English; therefore, we constrained it to the defined types and requested that it adhere to the regulations in order to avoid translation. This enabled us to match the extracted candidate PHI with the input text and determine their spans for the purpose of performance evaluation.
4. Retrieval message: We provided the text “PHIs:” to instruct the model to generate results to complete the task.

The experiment was conducted from February 20, 2023, to April 30, 2023, using the GPT-3.5 model (version from February 13, 2023) with temperature and top_*P* values set at 0.5. As we used the zero-shot in-context learning setting, we observed that ChatGPT could generate different responses for the same prompt. Thus, we sent the same prompt 3 times to collect all PHI candidates and retained the PHI that appeared at least twice as the final results. The decision to send the prompt 3 times aligns with previous work [50,51], which recommended this practice to ensure the stability of the responses.

### Evaluation Metrics and Analysis Methods Used in This Study

#### Deidentification Performance Evaluation

Performance of the developed methods was measured by standard metrics used in previous deidentification tasks [18] including recall and precision. The overall performance was assessed using the micro-F_1_ measure, a weighted harmonic mean of precision and recall. We further report the macro-F_1_ measure as the unweighted mean of the F-scores calculated per PHI type. For the evaluation of the results of the proposed ChatGPT-based deidentification framework, we applied the approximate match criterion [52] to determine whether the recognized boundary matched with the human annotated span.

In addition, to gain more insights into the impact of the code-mixing level on PHI recognition, we further categorized sentences according to their CMI levels and estimated the corresponding sentence error rates, which were computed by dividing the total number of wrongly predicted sentences by the total number of sentences in each CMI range. A sentence was considered incorrectly predicted if any of its tokens were labeled incorrectly.

#### Code-Mixing Level Evaluation

To measure the level of complexity and mixing in our corpus, we applied the CMI defined in equation (1) to calculate the average CMI for a specific language *L*, termed AvgCMI*_L_*. In this study, a sentence *s* was classified as either English-dominated or Chinese-dominated according to the dominating tokens present in the sentence. AvgCMI*_L_* was defined as follows:



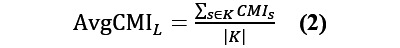



where *L* is either the English or Chinese language and *K* indicates the set of *L*-dominated sentences in our corpus. Note that the calculation of AvgCMI*_L_* includes the sentences with a CMI of zero. Finally, the CMI for the corpus (CMIC) was defined as follows:



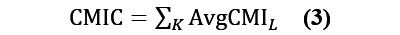



#### Methods for Diagnosing and Interpreting the Outputs of the Developed Models

According to our experimental findings, the primary factor affecting PHI recognition performance was the problem of ambiguity. This problem arises when the identified words or phrases can be categorized under more than one PHI type. For example, “八里 (Bali)” is a suburban district in northwestern New Taipei, Taiwan, but it can also refer to “八里療養院 (Bali psychiatric center)” in sentences such as “Informant: Discharge note from 八里.” To interpret the produced representations and analyze the ambiguity issues encountered, we used the t-Distributed Stochastic Neighbor Embedding (t-SNE) [53] method to project the hidden representations of PHI tokens from the last transformer layer of the developed models.

In our subsequent experiments, we noticed that EN-BERT can recognize the target PHI even if the information is described in Chinese characters or is mentioned in sentences that consist of English-Chinese or Chinese characters only. Moreover, we found that all BERT-based models could recognize a target PHI as a candidate, even if its encoding is replaced with “UNK” (unknown word). For instance, in the sentence “... follow-up in Dr. 張家明’s OPD,” where “張家明” is recognized as “Doctor,” replacing the encoding of “張家明” in the sentence with “UNK” did not affect the ability of BERT-based models to recognize it as candidate PHI of “Doctor.”

To comprehend the decision-making mechanism, we probed the results of the developed models by applying the input reduction method [54] and interpreted it using the attention-head view (AHV) to visualize the attention patterns produced by the models’ attention heads in the last transformer layer. The input reduction method removes as many words as possible based on the calculated gradient values, without altering a tag’s prediction. The AHV visualization was generated by the BERTViz tool [55].

## Results

### PHI Distribution and Code-Mixing Level Estimation

Given the code-mixing characteristics of our corpus, we categorized sentences in our data set based on their code-mixing characteristics into 4 groups: English only, Chinese only, a mixture of Chinese and English, and numeric/symbolic characters. The statistical information for PHI across the 4 groups is presented in Table 2. Overall, it is apparent that the distributions of different PHI types are significantly imbalanced. Without considering numeric/symbolic sentences, the most frequent type of sentences was English only, followed by code-mixed and Chinese only. The “Date” type was most frequently observed in numeric/symbolic sentences, while the “Age” type was most commonly noted in English-only sentences. The “Name” and “Profession” types were most frequently observed in code-mixed sentences, and the “Location” type was evenly distributed across English-only, Chinese-only, and code-mixed sentences. Finally, the “ID” type was most commonly found in Chinese-only sentences.

The AvgCMI_English_, AvgCMI_Chinese_, and CMIC estimated for the training set, test set, and entire corpus are presented in Multimedia Appendix 3, which highlight the writing convention in Taiwan’s medical records, with the majority of records being written in English. Another interesting writing convention observed was that code mixing was more frequent in Chinese-dominated sentences than in English-dominated sentences. The significant difference between AvgCMI_English_ and AvgCMI_Chinese_ highlighted that the frequency of Chinese tokens occurring in English-dominated sentences is much less than the frequency of English tokens occurring in Chinese-dominated sentences. The CMIC of our corpus was 22.21, which is significantly higher than the CMIC of English-Bangla (approximately 5.15) and Dutch-Turkish (approximately 4.13) data sets [45].

**Table 2:** Protected health information statistics for the 4 sentence groups (English-only sentences, Chinese-only sentences, mixed Chinese-English sentences, and numeric or symbolic sentences).

Protected health information type		English-only sentences, n		Chinese-only sentences, n		Chinese-English mixed sentences, n		Numeric or symbolic sentences, n		Entire corpus, n	
		Training set	Test set	Training set	Test set	Training set	Test set	Training set	Test set	Training set	Test set
Date^a^		12,436	2346	2146	458	4530	919	19,850	3724	38,962	7447
Age^a^		2444	533	21	5	639	128	0	0	3104	666
**Name^a^**		100	18	273	44	1339	240	0	0	1712	302
	Patient	9	9	106	12	42	11	0	0	157	32
	Person	8	3	6	1	128	32	0	0	142	36
	Doctor	83	6	161	31	1169	197	0	0	1413	234
**Location^a^**		5030	1014	4812	1108	5330	1132	4	3	15,176	3257
	Named location	17	10	11	3	271	69	0	0	299	82
	Nationality	21	7	3	1	17	10	0	0	41	18
	Region	6	11	0	0	10	1	0	0	16	12
	Country	355	52	6	5	171	44	0	0	532	101
	City	196	39	26	6	510	101	0	0	732	146
	Hospital	1552	266	73	11	1915	393	0	0	3540	670
	Department	1616	391	2278	542	762	111	0	0	4656	1044
	Room	1007	182	1200	267	248	53	4	3	2459	505
	Number	0	0	1162	256	25	1	0	0	1187	257
	School	28	6	15	2	642	159	0	0	685	167
	Generic location	218	45	37	15	708	173	0	0	963	233
	Market	12	3	1	0	50	17	0	0	63	20
Profession^a^		575	107	82	9	1566	449	0	0	2223	565
**ID^a^**		54	0	192	26	197	5	7	0	449	31
	ID number	43	0	98	11	170	2	7	0	318	13
	Medical record	1	0	92	14	19	3	0	0	112	17
Total number of sentences		20,639	4018	7526	1650	13,604	2873	19,861	3727	61,630	12,268

^a^Coarse-grained protected health information.

### Deidentification Performance Comparison

The performance comparison of the developed deidentification methods is shown in Table 3, where the DBA indicates the dictionary-based baseline. The remaining configurations include monolingual BERT models (EN-BERT and CH-BERT) and multilingual BERT models (M-BERT and TM-BERT).

The results showed a significantly lower performance of the DBA, highlighting that the dictionary collected from the training set is insufficient to provide reliable deidentification results. To be specific, the micro- and macro-F-scores of the DBA were 0.13 and 0.16, respectively, while the micro- and macro-F-scores of BERT-based methods ranged from 0.90 to 0.94 and 0.63 to 0.79, respectively, which were significantly higher than those of the DBA. Among all BERT-based approaches, M-BERT outperformed the others in terms of both micro- and macro-F-scores. CH-BERT demonstrated a performance comparable to that of M-BERT on various PHI types such as “Name” and “Location.” The micro- and macro-F-scores of TM-BERT were higher than those of EN-BERT but lower than those of CH-BERT and M-BERT, suggesting that translating code-mixed sentences into monolingual text does not provide advantages. TM-BERT’s lower performance was mainly due to translation errors that can cause syntactic or semantic issues during fine-tuning. Some words like abbreviations (“NKUST”) and Chinese names that are PHI mentions cannot be accurately translated. Even context words could alter their meaning, for example, the text “occupation: 英語補習班老師” (Profession: English tutoring teacher) was translated as “佔領: 英語補習班老師” (Domination: English tutoring teacher).

To gain a deeper understanding of the ability of PLMs to recognize different types of PHI mentioned in sentences with different levels of language mixing, we categorized the sentences in our corpus into 3 distinct sentence categories: English-only, Chinese-only, and Chinese-English mixed. We then evaluated the performance of the developed methods for each category. The results are presented in Table 4, focusing on the comparison between EN-BERT, CH-BERT, and M-BERT only. We observed that the distribution of different PHI types varied across the 3 sentence categories, suggesting that the performance of the developed models may be influenced by the prevalence of certain PHI types in their respective sentence categories. Specifically, we found that numeric PHI types, such as “Date” and “Age,” were more prevalent in English-only sentences, while nonnumeric PHI types, including “Name” and “Profession,” were more frequent in code-mixed sentences. This finding explains why EN-BERT performed similarly to multilingual and translation models on numeric PHI types but much worse on nonnumeric types. Additionally, the generally high performance for numeric PHI types indicates that mixing Chinese and English in the same sentence has little effect on their recognitions. Detailed results can be found in Multimedia Appendix 3.

In contrast, the “ID” PHI type occurred most frequently in the Chinese-only sentences, but all BERT-based models performed well in recognizing them. The “Location” PHI type was equally present in all 3 categories, but certain fine-grained types, such as “Region” and “Nationality,” were more challenging due to their limited training instances. Overall, the results showed that M-BERT outperformed the other models in the 3 sentence categories based on the metric of micro-F-score, while CH-BERT had the best performance in terms of macro-F-score in Chinese-only and code-mixed sentences. EN-BERT exhibited comparable performance with M-BERT in English-only sentences, but its performance was inferior to that of CH-BERT and M-BERT in Chinese-only and code-mixed sentences, indicating that these types of sentences are more challenging for EN-BERT. In contrast, both CH-BERT and M-BERT exhibited less effect of the code-mixing issue in their recognition of PHI in code-mixed sentences.

**Table 3:** Performance comparison for different methods on the test set.

Protected health information type			DBA^a^						EN-BERT^b,c^							CH-BERT^d^							M-BERT^e^							TM-BERT^f^				
			P^g^		R^h^		F^i^		P		R		F		P			R		F		P			R		F		P			R		F
Date			0.07		0.19		0.11		0.97		0.98^j^		0.98^j^		0.97			0.98^j^		0.97		0.98^j^			0.98^j^		0.98^j^		0.97			0.97		0.97
Age			0.14		0.16		0.15		0.95^j^		0.97		0.96^j^		0.94			0.98^j^		0.96^j^		0.94			0.97		0.95		0.93			0.97		0.95
**Name**			0.06		0.14		0.09		0.62		0.47		0.51		0.82			0.70^j^		0.74		0.87^j^			0.68		0.75^j^		0.73			0.59		0.64
	Patient	0.04		0.09		0.05		0.59		0.41		0.48		1.00^j^			0.59^j^		0.75^j^		1.00^j^			0.59^j^		0.75^j^		0.94			0.59		0.72	
	Person	0.04		0.11		0.06		0.36		0.11		0.17		0.54			0.58^j^		0.56		0.66^j^			0.53		0.56^j^		0.30			0.25		0.27	
	Doctor	0.11		0.22		0.15		0.90		0.88		0.89		0.92			0.92		0.92		0.94			0.92		0.93		0.95^j^			0.93^j^		0.94^j^	
**Location**			0.14		0.20		0.15		0.64		0.56		0.58		0.79^j^			0.69		0.72		0.78			0.74^j^		0.75^j^		0.67			0.71		0.69
	Named location	0.01		0.01		0.01		0.34		0.26		0.29		0.64			0.37		0.47		0.61^j^			0.57^j^		0.59^j^		0.34			0.34		0.34	
	Nationality	0.43		0.14		0.21		0.67^j^		0.19		0.30		0.44			0.52		0.48		0.53			0.48		0.50		0.48			0.53^j^		0.50^j^	
	Region	0.00		0.00		0.00		0.67^j^		0.33^j^		0.44^j^		0.67^j^			0.17		0.27		0.29			0.17		0.23		0.20			0.17		0.18	
	Country	0.25		0.33		0.28		0.76		0.75		0.75		0.89			0.89		0.89		0.91^j^			0.90^j^		0.91^j^		0.87			0.86		0.87	
	City	0.21		0.52		0.30		0.58		0.59		0.58		0.86			0.74		0.79		0.86^j^			0.77^j^		0.82^j^		0.77			0.73		0.75	
	Hospital	0.35		0.47		0.40		0.84		0.85		0.84		0.89			0.94		0.91		0.93^j^			0.94^j^		0.94^j^		0.90			0.90		0.90	
	Department	0.24		0.35		0.29		0.90		0.87		0.88		0.92			0.88		0.90		0.93^j^			0.90^j^		0.92^j^		0.90			0.87		0.89	
	Room	0.04		0.15		0.06		0.87		0.92		0.89		0.89			0.94		0.91		0.91^j^			0.94^j^		0.92^j^		0.77			0.94		0.85	
	Number	0.05		0.08		0.06		0.99		1.00^j^		0.99		0.99			1.00^j^		0.99		0.99			1.00^j^		1.00^j^		1.00^j^			1.00^j^		1.00^j^	
	School	0.01		0.01		0.01		0.64		0.66		0.65		0.91^j^			0.82		0.86		0.85			0.87^j^		0.86^j^		0.76			0.82		0.79	
	General business	0.09		0.16		0.11		0.42		0.31		0.36		0.66			0.64		0.65		0.70^j^			0.73^j^		0.71^j^		0.61			0.61		0.61	
	Market	0.09		0.15		0.11		0.00		0.00		0.00		0.67			0.40		0.50		0.79^j^			0.55		0.65^j^		0.45			0.75^j^		0.57	
Profession			0.09		0.22		0.12		0.46		0.43		0.44		0.66			0.73		0.69		0.78^j^			0.76^j^		0.77^j^		0.59			0.72		0.65
**ID**			0.34		0.31		0.32		0.75		0.96		0.83		0.79			1.00^j^		0.86		0.81^j^			1.00^j^		0.90^j^		0.77			1.00^j^		0.85
	ID number	0.67		0.62		0.64		0.55		0.92		0.69		0.57			1.00^j^		0.72		0.65^j^			1.00^j^		0.79^j^		0.53			1.00^j^		0.70	
	Medical record	0.00		0.00		0.00		0.94		1.00^j^		0.97		1.00^j^			1.00^j^		1.00^j^		1.00^j^			1.00^j^		1.00^j^		1.00^j^			1.00^j^		1.00^j^	
Micro-Average			0.09		0.22		0.13		0.91		0.90		0.90		0.93			0.93		0.93		0.94^j^			0.94^j^		0.94^j^		0.90			0.92		0.91
Macro-Average			0.15		0.20		0.16		0.67		0.62		0.63		0.80			0.75		0.76		0.81^j^			0.78^j^		0.79^j^		0.71			0.75		0.72

^a^DBA: dictionary-based approach.

^b^BERT: Bidirectional Encoder Representations from Transformers.

^c^EN-BERT: BERT pretrained on English corpora.

^d^CH-BERT: BERT pretrained on simplified and traditional Chinese corpora.

^e^M-BERT: BERT pretrained on Wikipedia corpora from 104 languages.

^f^TM-BERT: M-BERT fine-tuned on the translated deidentification corpus.

^g^P refers to precision.

^h^R refers to recall.

^i^F refers to F1-score.

^j^The highest score among different methods.

**Table 4:** Comparison of performance between EN-BERT, CH-BERT, and M-BERT in English-only, Chinese-only, and Chinese-English mixed sentences.

PHI^a^ type		English-only sentences					Chinese-only sentences					Chinese-English mixed sentences			
		EN-BERT^b,c^	CH-BERT^d^	M-BERT^e^	PHI, n^f^	EN-BERT		CH-BERT	M-BERT	PHI, n^f^	EN-BERT		CH-BERT	M-BERT	PHI, n^f^
Date^g^		0.96^h^	0.94	0.95^h^	2346^h^	0.98		0.97	0.99^h^	458^h^	0.93		0.93	0.94^h^	919^h^
Age^g^		0.97^h^	0.96	0.96	533^h^	0.67		0.91^h^	0.73	5^h^	0.94		0.97^h^	0.95	128^h^
**Name^g^**		0.24^h^	0.24^h^	0.24^h^	18^h^	0.38		0.54^h^	0.53	44^h^	0.54		0.80^h^	0.78	240^h^
	Patient	0.00	0.00	0.00	9	0.64		1.00^h^	1.00^h^	12	0.50		0.78	0.88^h^	11
	Person	N/A^i^	N/A	N/A	0	0.00		0.00	0.00	1	0.18		0.64^h^	0.48	32
	Doctor	0.94^h^	0.94^h^	0.94^h^	9	0.49		0.61^h^	0.60	31	0.94		0.97	0.98^h^	197
**Location^g^**		0.66	0.61	0.70^h^	1014^h^	0.36		0.87^h^	0.83	1108^h^	0.37		0.66^h^	0.65	1132^h^
	Named location	0.47	0.25	0.62^h^	10	0.00		0.40^h^	0.40^h^	3	0.28		0.53^h^	0.52	69
	Nationality	0.67^h^	0.38	0.67^h^	7	0.00		1.00^h^	1.00^h^	1	0.00		0.86^h^	0.78	10
	Region	0.47	0.55^h^	0.50	11	N/A		N/A	N/A	N/A	0.00		0.00	0.00	1
	Country	0.87	0.85	0.95^h^	52	0.25		1.00^h^	1.00^h^	5	0.63		0.94^h^	0.88	44
	City	0.74	0.72	0.77^h^	39	0.22		1.00^h^	0.92	6	0.53		0.85^h^	0.82	101
	Hospital	0.93^h^	0.92	0.93^h^	266	0.21		0.80	0.83^h^	11	0.79		0.92	0.93^h^	393
	Department	0.82	0. 81	0.82^h^	391	0.95		0.96^h^	0.96^h^	542	0.68		0.76	0.77^h^	111
	Room	0.85	0.83	0.85^h^	182	0.97		0.98^h^	0.97	267	0.68		0.76^h^	0.76	53
	Number	N/A	N/A	N/A	N/A	0.99		1.00^h^	0.99	256	0.00		0.00	0.00	1
	School	0.71	0.25	0.83^h^	6	0.00		0.67^h^	0.50	2	0.65		0.92^h^	0.90	159
	Generic business	0.72^h^	0.59	0.70	45	0.00		0.89^h^	0.72	15	0.27		0.66	0.71^h^	173
	Market	0.00	0.50^h^	0.00	3	N/A		N/A	N/A	N/A	0.00		0.67	0.73^h^	17
Profession^g^		0.78	0.71	0.81^h^	107^h^	0.17		0.67^h^	0. 61	9^h^	0.36		0.70	0.75^h^	449^h^
**ID^g^**		N/A	N/A	N/A	N/A	0.90		0.90	0.91^h^	26^h^	0.60		0.90^h^	0.75	5^h^
	ID number	N/A	N/A	N/A	N/A	0.79		0.79	0.81^h^	11	0.33		0.80^h^	0.50	2
	Medical record	N/A	N/A	N/A	N/A	1.00^h^		1.00^h^	1.00^h^	14	0.86		1.00^h^	1.00^h^	3
Micro-F-score		0.92	0.90	0.92^h^	4018	0.93		0.96^h^	0.96^h^	1650	0.71		0.85	0.87^h^	2873
Macro-F-score		0.60^h^	0.54	0.60^h^	4018	0.47		0.83^h^	0.79	1650	0.48		0.74^h^	0.72	2873

^a^PHI: protected health information.

^b^BERT: Bidirectional Encoder Representations from Transformers.

^c^EN-BERT: BERT pretrained on English corpora.

^d^CH-BERT: BERT pretrained on simplified and traditional Chinese corpora.

^e^M-BERT: BERT pretrained on Wikipedia corpora from 104 languages.

^f^The number of training instances of the PHI type.

^g^Coarse-grained PHI.

^h^The highest F-score among the 3 methods.

^i^N/A: not applicable.

### Impact of the CMI Level on Deidentification Performance

**Figure 3:**
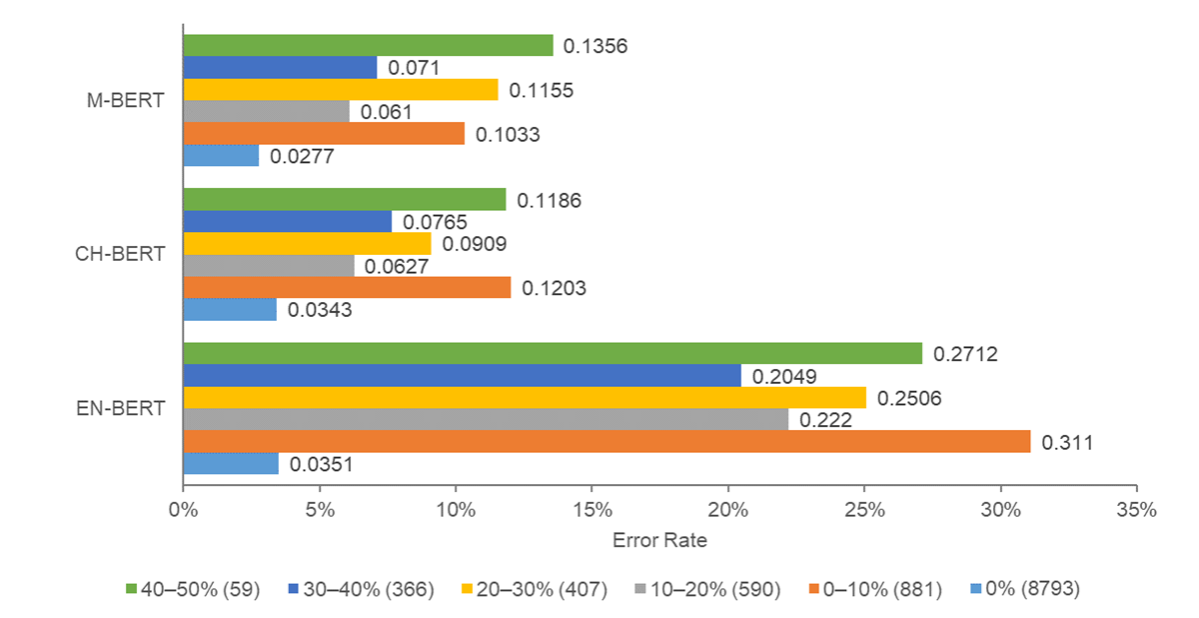
Sentence error rate in each code-mixing index range for the test set. The numbers in parentheses represent the number of incorrect examples for the corresponding code-mixing index range. CH-BERT: BERT pretrained on simplified and traditional Chinese corpora; EN-BERT: BERT pretrained on English corpora; M-BERT: BERT pretrained on Wikipedia corpora from 104 languages.

In this subsection, we report sentence error rates by dividing sentences in our corpus into 6 groups according to their CMI levels, as illustrated in Figure 3, to gain insights into the impact of the code-mixing level on PHI recognition. It is evident from the figure that EN-BERT has the highest error rate for all CMI ranges. This aligns with our earlier finding in the previous section demonstrating that EN-BERT struggles with recognizing PHI in code-mixed sentences. Although CH-BERT and M-BERT exhibited lower sentence error rates than EN-BERT, the error rates were still considerably higher for code-mixed sentences (CMI >0), indicating that the recognition of PHI in code-mixed sentences remains a challenging task for the 2 PLMs.

Further evidence supporting this conclusion can be found in Table 5, where we fine-tuned M-BERT on training instances from 1 sentence category and evaluated it on the test set categorized into the 3 categories. From the results, we observed that fine-tuning M-BERT with training instances from a specific sentence category can significantly improve its performance on that category. However, fine-tuning on the code-mixed training instances yielded the best results, which further enabled the model to effectively recognize PHI in both Chinese and English sentences. This finding emphasizes the significance of incorporating code-mixed training instances into the model’s training data.

**Table 5:** The “zero-shot” performance of BERT pretrained on Wikipedia corpora from 104 languages (M-BERT) for the 3 sentence categories in terms of micro-F-scores.

Evaluation/Fine-tuning	English-only	Chinese-only	Chinese-English mixed
English-only	0.93	0.42	0.57
Chinese-only	0.59	0.96	0.65
Chinese-English mixed	0.80	0.94	0.87

### Performance Comparison With ChatGPT

**Figure 4:**
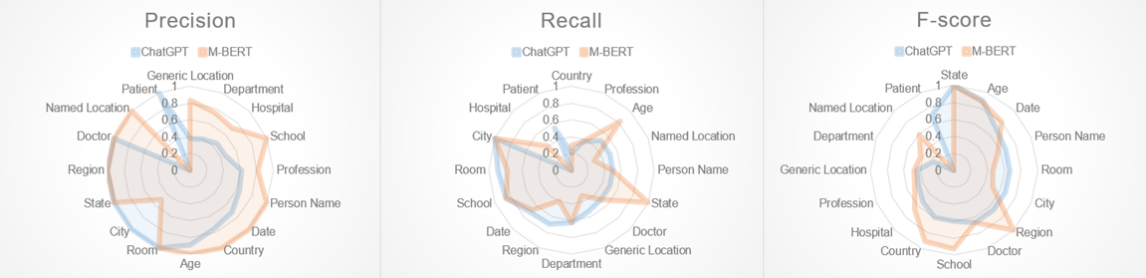
Performance comparison with the ChatGPT-based deidentification framework and M-BERT on the sampled test subset. M-BERT: BERT pretrained on Wikipedia corpora from 104 languages.

To assess the effectiveness of the proposed ChatGPT-based framework for recognizing PHI in our code-mixed corpus, we collected a subset of the original data that included sentences containing one or more PHI sets that were not recognized by any of the BERT-based models we developed. This subset was chosen to represent the most challenging cases of the compiled corpus while minimizing the risk of sensitive information exposure. Figure 4 shows the results, with M-BERT used for comparison.

Compared with M-BERT, the proposed ChatGPT-based framework achieved slightly better recall, but lower precision, leading to a lower micro-F-score in the zero-shot setting. Nonetheless, our framework presents an appealing approach to use LLMs for the deidentification task over standard-sized models like BERT. LLMs are notably easier to use and can be controlled through natural language prompts, requiring little to no machine learning expertise. For example, we found only 1 error case for the “Doctor” PHI that both BERT-based models and ChatGPT failed to recognize: “follow-up: (ydh/ptz).” However, we can enhance the prompt shown in Figure 2 by including a statement following the “DOCTOR NAME” definition that lists the abbreviation names of physicians in a hospital, directing ChatGPT to recognize “ptz” as a doctor name: “Here is a list of abbreviated physician names of our hospital. Remember and recognize them from the given text: 1. hjd, 2. ptz, 3. ctc.” This exemplifies the adaptability and flexibility of LLMs for deidentification tasks, making them particularly well-suited for real hospital environments. While the potential for using ChatGPT in deidentification is notable, the model is currently only accessible through an online application programming interface. This renders it unsuitable for use in a hospital setting, where patient data cannot be stored or transmitted to unauthorized external parties.

On the other hand, it crucially highlights the significance of carefully crafted prompts when working with ChatGPT. The constraint statements presented in Figure 1 play a pivotal role in guiding ChatGPT’s responses. When these constraints were removed, we observed that ChatGPT can generate responses that may be unpredictable and go beyond the desired control. Take the text “he was brought to Dr. 莊凱傑’s opd on 3/13 and haldol + anxicam 1 amp was given” as an example. Here, “莊凱傑” represents a Chinese name, which can be translated to “KAI-JIE ZHUANG.” Using the developed prompts without adding the constraint part, both the original and recently released ChatGPT 3.5 models (version from September 27, 2023) could potentially return responses like “MEDICATION: haldol, anxicam” and “DOSE: 1 amp.”

Another issue to consider is the translation of recognized PHI into a language other than that mentioned in the given text. The issue is more prominent in Chinese-majority code-mixed sentences, where the recognized Chinese PHI might be translated into the English counterparts. For example, in the sentence “education: 高中讀中和高中, 僅唸數個月就因跟不上而休學, 大學念正修夜間部商學” (education: high school-Zhonghe senior high school, dropped out after only a few months; College-Business in the night school at Cheng Shiu university), the Chinese PHI could be extracted as translated forms in English, which can lead to a problem in surrogate generation and affect the performance evaluation procedure.

Lastly, similar to the observations in previous work [50,51], we found that the practice of requesting ChatGPT to generate responses 3 times resulted in the improvement of the overall precision, recall, and F1-score by 0.055, 0.068, and 0.064, respectively. The strategy can effectively filter out noisy outputs generated by ChatGPT. For instance, even with the inclusion of constraint statements in our prompts, we noticed that ChatGPT occasionally generated responses that violated the defined constraints, introducing undesired entity types such as “MEDICATION,” “DOSE,” or “DIAGNOSIS.” By implementing the 3-query strategy, we could successfully eliminate these cases, ensuring the quality and relevance for our guideline of the generated content. Further details regarding the limitations for this strategy and its implications are provided in the limitations section.

## Discussion

### Ambiguity Issue Among PHI

We explored the issue of ambiguity in the classification of fine-grained PHI at the coarse-grained level. When fine-grained PHI is misclassified, it may belong to the same coarse-grained PHI or different ones. Misclassifying it under the same coarse-grained PHI is generally more acceptable since fine-grained PHI belonging to the same coarse-grained category tends to share similar semantic characteristics. To quantify the extent of ambiguity, we calculated the micro-F-scores at the coarse-grained level, treating an incorrect recognition of a fine-grained PHI type as a true positive if it belongs to the same coarse-grained PHI type as the corresponding gold annotation. Our results (detailed in Multimedia Appendix 4) showed that intratype ambiguities are more prevalent than intertype ambiguities in “Name” and “Location” PHI types.

**Figure 5:**
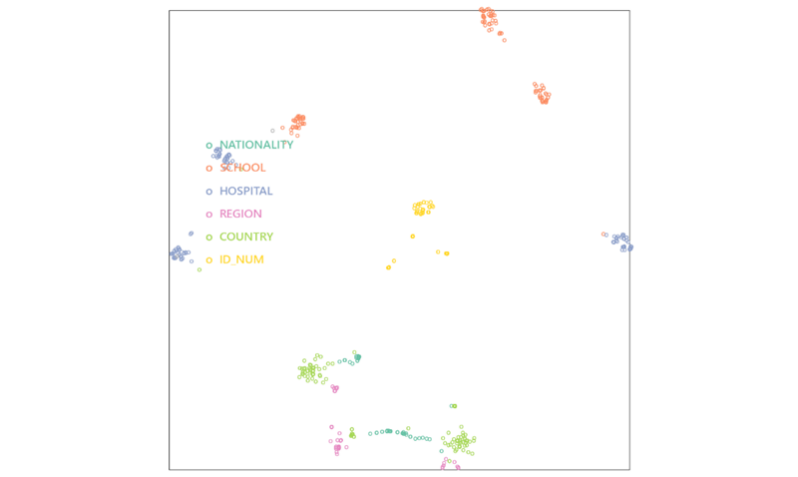
t-Distributed Stochastic Neighbor Embedding visualization of the 5 fine-grained “Location” protected health information (PHI) types and the “ID” PHI type for M-BERT. M-BERT: BERT pretrained on Wikipedia corpora from 104 languages.

To gain insights into the level of ambiguity among the “Location” fine-grained types, we visualized the representations generated by M-BERT for different “Location” PHI types through t-SNE. The resulting plot (Figure 5) revealed that the clusters of “Nationality,” “Country,” and “Region” are intertwined, while the cluster of “ID” is notably separated from the others. The high ambiguity may be due to the limited number of training instances, as the number of training instances is imbalanced among PHI as illustrated in Table 2. As a result, all BERT-based models yielded better F-scores for “Country” than for “Nationality” and “Region.”

On the other hand, the “ID” PHI type had a perfect mapped recall, indicating that all of the coarse-grained PHI could be recognized by the developed models. The confusion matrix shown in Figure S1 in Multimedia Appendix 4 reveals only 1 ambiguous case where M-BERT misclassified the “Location” PHI type as the “ID” PHI type. This occurred in the sentence “She visited psychiatric clinic of 804 then but have neither regular follow-up nor fair drug compliance,” where “804” refers to the Military Taoyuan General Hospital (804 is the army number assigned to the hospital), but the model recognized it as “ID” PHI. This example demonstrates one of the most challenging cases where the model failed at recognition, even with the recall-oriented ChatGPT framework. To correctly classify the instance, the model requires additional domain knowledge. We attempted to use ChatGPT to determine whether it knows that “804 hospital” refers to the military hospital, but it replied that “804 hospital” was not a common name or official name for the hospital. However, a quick Google search revealed that the website of the hospital was the first suggested link, indicating the importance of external knowledge sources for accurate classification [56].

### OOV Case Study to Interpret the Models’ Decisions

**Figure 6:**
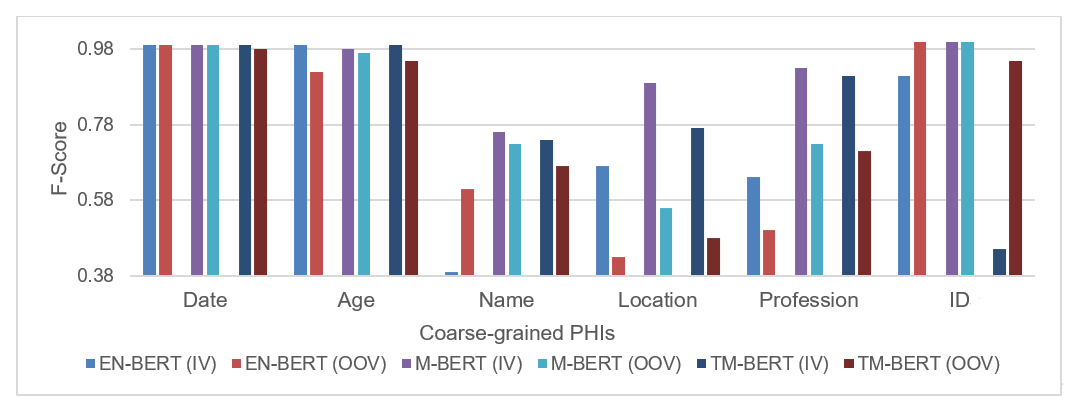
F-scores of coarse-grained protected health information (PHI) for tokens belonging to in-vocabulary (IV) and out-of-vocabulary (OOV) in the test set. EN-BERT: BERT pretrained on English corpora; M-BERT: BERT pretrained on Wikipedia corpora from 104 languages; TM-BERT: M-BERT fine-tuned on the translated deidentification corpus.

This section delves into how the BERT-based models use regularity and mention coverage, as outlined in the Introduction section, during their recognition process. First, we estimated the OOV rates on the test set to determine the mention coverage rate of our corpus. The estimated rates were 0.356, 0.953, 0.686, 0.876, 0.696, and 0.290 for the “Date,” “Age,” “Name,” “Location,” “Profession,” and “ID” categories, respectively. OOV was defined as a mention containing at least one word not present in the vocabulary compiled from the training data set. The inadequate performance of the DBA along with the high OOV rate suggests that the effect of the mention coverage in our corpus is limited. To analyze the effect, we re-estimated the performance of coarse-grained PHI for tokens belonging to in-vocabulary (IV) and OOV using EN-BERT, M-BERT, and TM-BERT in Figure 6. More detailed results are available in Multimedia Appendix 4.

**Figure 7:**
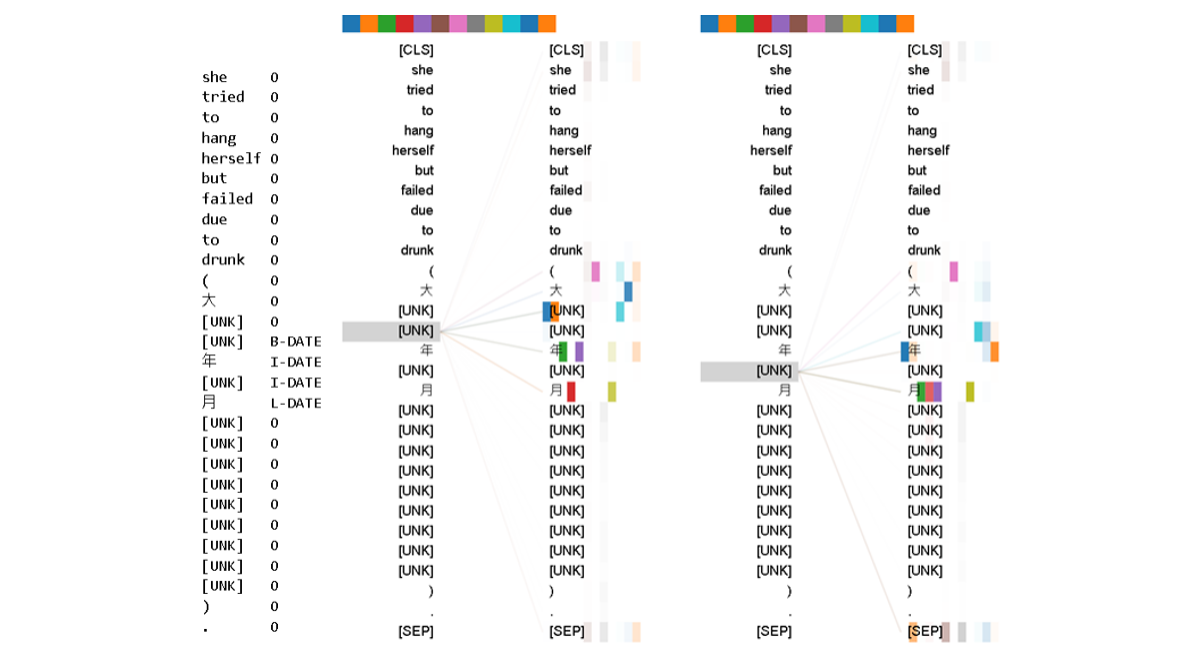
The attention-head view for the following code-mixed sentence: “she tried to hang herself but failed due to drunk (大約前年五月開始不斷有死的念頭)” with the tokens “[UNK]” before/after “年 (year)” selected. The English translation for the Chinese text is “she had suicidal thoughts since around May of two years ago.” [UNK] is the token for unknown word. [CLS] is a classification token and is added to the beginning of every sentence. [SEP] is a separation token inserted in between 2 sentences.

The presented results reveal that the developed models have different levels of tolerance for OOV issues when recognizing different PHI types. Interestingly, we observed that the EN-BERT model achieved indistinguishable high F-scores for IV and OOV tokens for the “Date” PHI type. This is notable since not all Chinese characters used in the “Data” PHI are presented in the EN-BERT vocabulary. For example, the numeric value “六” (six) is not available as shown in Table 6. We believe that the presence of those characters in the extensive training data enabled EN-BERT to leverage the naming regularity of the “Date” PHI and improve its ability to identify occurrences of these entities in Chinese-only and code-mixed sentences. Figure 7 illustrates how EN-BERT’s attention mechanism can use specific patterns in text, such as “年月日,” for recognizing “Date” PHI, even when the majority of the Chinese tokens are mapped to [UNK] (unknown word) by the EN-BERT tokenizer. The model’s attention was primarily drawn to the pattern words “年” and “月,” allowing it to successfully assign labels to the respective unknown words.

**Figure 8:**
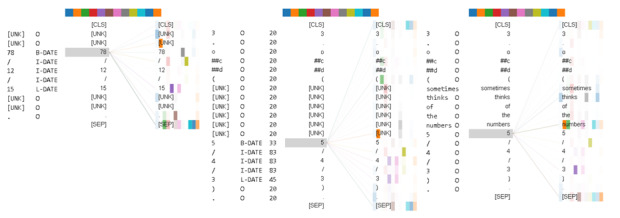
The first and second attention-head views have been generated for EN-BERT when applied to process the following sentences: “他在 78/12/15 求診” (sampled from the training set; the English translation is “He went to see a doctor on 78/12/15”; “78/12/15” is annotated as “Date” PHI) and “3. ocd (不時想到數字5/4/3/8)” (sampled from the test set; the English translation is “sometimes thinks of the numbers 5/4/3/8”). The third view has been generated for EN-BERT when processing the translated text of the second sentence. [UNK] is the token for unknown word. [CLS] is a classification token and is added to the beginning of every sentence. [SEP] is a separation token inserted in between 2 sentences. EN-BERT: BERT pretrained on English corpora; PHI: protected health information.

However, the model’s overreliance on pattern words and strong name regularities could lead to misclassification. Figure 8 illustrates an example of the false-positive case generated by EN-BERT. The model has been trained to recognize the first likely token for “Date” PHI by considering its surrounding tokens, including previous unidentified tokens, the forward slash (/), and the final token representing the end of the “Date” PHI. However, this mechanism caused the model to incorrectly extract the text “5/4/3” as “Date” PHI. The third AHV displayed in Figure 4 illustrates that when the previous tokens are not unknown, the model focuses more on the preceding token (a number) and gives less attention to the pattern tokens (“/”), which helps prevent the occurrence of false positives.

On the other hand, the “Age,” “Location,” and “Profession” PHI types exhibited higher IV F-scores compared to their OOV scores. Specifically, the “Age” PHI type demonstrated an almost perfect IV F-score and slightly lower OOV score across all 3 models. After examining the outcomes of the developed models, we arrived at a conclusion similar to that of the “Date” PHI. The models could distinguish the “Age” PHI type in various code-mixing–level sentences because of the consistent naming pattern. There were only a few cases in which the developed models failed. For instance, in the sentence, “occupation: 會計18y->美容師3y->保險電話銷售人員->未上市股票電話銷售員->賣雞排 (2223.3-2224.9),” only CH-BERT was able to correctly avoid classifying “18” as “Age” PHI, while the other BERT-based models and the developed ChatGPT framework recognized “18” as “Age” PHI.

**Figure 9:**
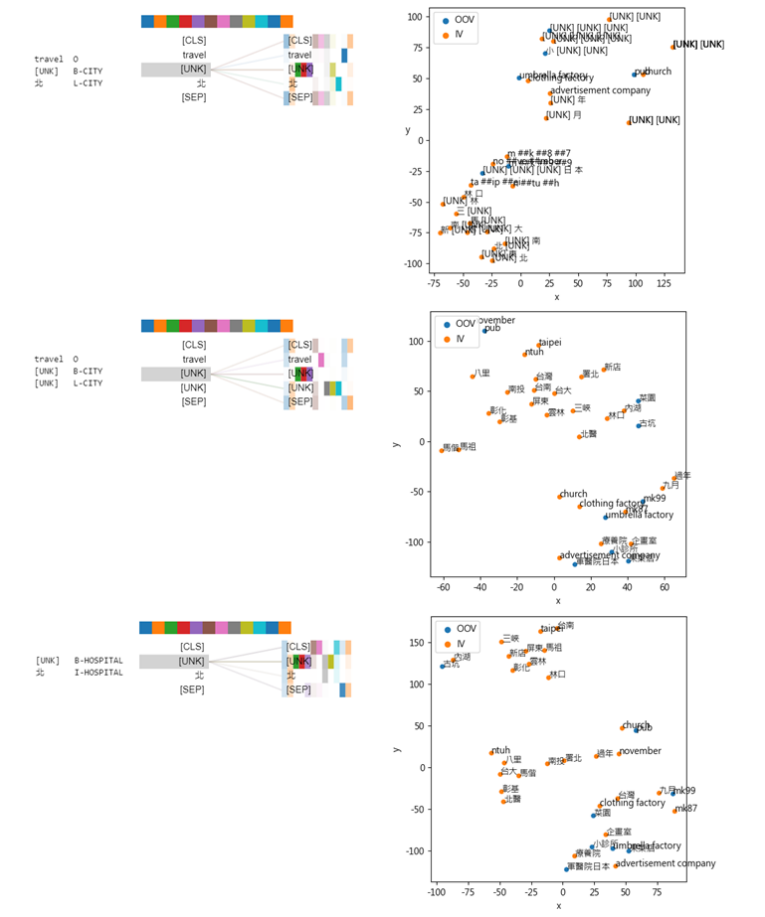
The left part shows the attention-head views (AHVs) of the input reduction probing for the input text “travel history: [PHI] last week.” The first column of the AHVs comprises 3 rows, wherein the PHI includes “台北” and “恆春.” We also present the result of the input “台北” in the last row of the first column in the view. The right part shows the t-Distributed Stochastic Neighbor Embedding visualizations of EN-BERT, M-BERT without fine-tuning, and M-BERT with fine-tuning from top to bottom for randomly sampled in-vocabulary (IV) and out-of-vocabulary (OOV) mentions of the 3 fine-grained “Location” PHI types including “Named location,” “Generic location,” and “Hospital.” For comparison, we also include 5 “Date” PHI instances including “過年” (New Year), “November,” “九月” (September), “mk87,” and “mk99” (where “mk” is a transliteration of “民國,” which refers to the Republic of China calendar). [UNK] is the token for unknown word. [CLS] is a classification token and is added to the beginning of every sentence. [SEP] is a separation token inserted in between 2 sentences. PHI: protected health information; EN-BERT: BERT pretrained on English corpora; M-BERT: BERT pretrained on Wikipedia corpora from 104 languages.

In contrast, the “Location” and “Profession” PHI types exhibited lower IV F-scores and apparently lower OOV F-scores. To investigate the discrepancies, we probed the results of the developed model by applying input reduction. We took the input sentence “travel history: [PHI] last week” as an example to summarize the observations as follows. First, we noticed that for EN-BERT, the presence of unknown tokens in the input sentence resulted in heavy reliance on the regularity of entity naming and contextual patterns for effective generalization over unseen mentions or mentions that contain unknown words. In the left part of Figure 9, the AHV represents the reduced interpretation of the 2 “City” PHI inputs “台北” (Taipei) and “恆春” (Hengchun) that contain unknown words. From the view, we can see that the preceding token, “travel,” provides a strong indication for EN-BERT to recognize “City” PHI. The word “台北” (“[UNK]北” for EN-BERT) was considered as “Hospital” PHI, indicating that the model learned to memorize the most frequent label assigned to the IV word, such as “署/[UNK]北” hospitals, in the training set.

Second, in addition to the naming regularity and contextual patterns, CH-BERT and M-BERT can leverage their representation for the given token sequence to assign the corresponding labels better. This is evident from the right part of Figure 5 in which we can see after fine-tuning, M-BERT was able to improve its representation of “Hospital” PHI entities, such as “ntuh,” “台大” (National Taiwan University Hospital), and “署北” (Taipei Hospital), as well as “Date” PHI entities, such as “九月” (September) and “mk87,” by clustering them in closer proximity to each other. Despite being OOV entities, OOV PHI, such as “mk99,” “菜園” (vegetable garden), “軍醫院” (military hospital), and “小診所” (small clinic), were still represented in close proximity to the corresponding IV PHI, such as “mk87,” “clothing factory,” and “療養院” (nursing home). However, “Profession” PHI and some of the fine-grained “Location” PHI types, such as “Generic location,” lacked the regularity of their naming conventions, which posed a challenge for accurate recognition. Moreover, informal language, typographical errors, ambiguity issues, and limited training instances for fine-grained PHI, such as “Region” and “Market,” further complicate the task of correctly identifying these OOV PHI mentions.

To mitigate the aforementioned OOV issue, we highlight several promising research directions. The first direction is data augmentation. During the pretraining phases, BERT-based models can benefit from automatically generating synthetic training examples that include PHI. This can be accomplished by leveraging LLMs to create data of reasonable quality [57]. This approach, similar to data augmentation, should help improve the model’s robustness when handling different contexts and OOV PHI. The second direction is vocabulary expansion. For specific categories like “Location” and “Profession,” where we observed lower F-scores on OOV PHI, traditional dictionary expansion methods [58] can be applied to augment BERT’s vocabulary. This approach empowers the model to recognize and process OOV and UNK words more effectively. The third direction is knowledge graph integration [59]. This approach enriches the contextual information available to the model, providing additional context and semantics to assist in recognizing OOV or UNK PHI and thereby improving overall performance.

**Table 6:** Examples of Chinese characters related to the “Date” protected health information listed in the BERT pretrained on English corpora (EN-BERT) vocabulary.

Chinese character	Example “Date” PHI^a^
清、明、中、秋	清明節、中秋節
一、二、三、四、五、八、十、月、日、年、上、下、年、西、星、民、國	三月八日、上周五、下周一、西元2321年、星期五、民國一一二年、今年十一月

^a^PHI: protected health information.

### Limitations

While our study has made significant contributions to the field of clinical text deidentification in code-mixed languages, it is important to acknowledge some limitations. First, despite the successes achieved, M-BERT, our best-performing model, has some limitations. Notably, it exhibits intratype ambiguities in certain PHI, such as “Location,” likely due to the limited number of training instances. Second, M-BERT yields decreased performance for OOV terms compared to IV terms in specific PHI categories, such as “Profession” and “Location.” Third, our study was based on a Chinese-English code-mixed corpus from Taiwan, which has its unique writing conventions and style. As a result, the fine-tuned models may not be entirely applicable to other bilingual or multilingual code-mixed corpora from different countries.

Another notable limitation is related to the normalization of mixed sentences into their dominant word-usage language based on the CMI. In the training process for TM-BERT, we relied on an existing translation model as a means to normalize code-mixed text. While this strategy was applied to alleviate the code-mixing challenge, it is crucial to note that translation errors because of the limited context can introduce syntactic and semantic issues during fine-tuning, which can, in turn, impact the model’s overall performance. This limitation underscores the need for further research to develop more sophisticated and accurate code-mixed text normalization techniques, as explored previously [60].

In the context of the proposed ChatGPT-based deidentification framework, we acknowledge that the framework’s performance is sensitive to several factors, including the crafted prompts, the number of query times applied for the majority voting strategy, and the specific model version used. For example, we observed that when using the recently released model (version from September 27, 2023) for recognizing PHI, it experienced a higher frequency of failures due to its reluctance to process sensitive content, as compared to the version we employed in our experiments. To address this limitation, it is needed to incorporate new specific constraints, such as “None of your responses will contain ‘I'm sorry,’ ‘I apologize,’ ‘I'm sorry, but I can’t assist with that request,’ or similar.” These constraints were introduced to mitigate potential issues, but they also shed light on another limitation. As ChatGPT is subject to continuous updates by OpenAI, the specific model version we used for our study may not always reflect the latest advancements in performance. This dynamic nature of the model versions could impact the generalizability of our results and the application of the framework in evolving contexts. We acknowledge this as a potential limitation, and it is an area where ongoing research and adaptation will be essential to keep pace with model improvements and changes.

Lastly, while our decision to use the prompt 3 times is consistent with prior methodologies [50,51], we acknowledge that the specific number of repetitions can be subject to experimentation and may depend on the context and objectives of the task. Future research could delve into the selection of repetitions and its influence on result distribution to gain a deeper understanding of this aspect.

### Conclusions

We developed the first-ever code-mixed clinical deidentification corpus, which exhibits a higher CMIC value compared to other known code-mixed data sets, suggesting that Chinese-English code-mixed clinical writing style is extensively used in the presented data set. We found that different PHI types had preference in their occurrence within the sentence categories, with “Date” and “Age” PHI types more common in English-only sentences, and “Name” and “Profession” PHI types more common in code-mixed sentences.

Our hypothesis that PLMs rely on naming regularity to recognize PHI was supported by our experimental results. Additionally, the results indicated that PLMs like M-BERT also use their learned representations to enhance their classification performance. We found that the fine-tuned M-BERT models outperformed the other approaches in most PHI types. We also observed that fine-tuning with code-mixed training instances is critical for significantly improving performance on the code-mixed data set. This finding emphasizes the significance of incorporating code-mixed training instances into the model’s training data. Our analysis revealed that TM-BERT yielded lower performance, suggesting that machine translation is not required for M-BERT in addressing the challenges posed by code mixing owing to the error-prone nature of machine-translated sentences.

We conclude that the LLM-based deidentification method is a feasible and appealing approach, which requires little to no machine learning expertise, and can be controlled and enhanced through natural language prompts. The experience of engineering our prompt also emphasizes the crucial role of carefully crafted prompts to avoid unwanted output. Further research could explore the augmentation of PLMs and LLMs with external knowledge to improve the strength in recognizing rare PHI and could incorporate experiments using the recently released GPT-4, which has been reported to demonstrate significant improvements in many tasks. However, the use of such a method in the hospital setting requires careful consideration of data security and privacy concerns.

## Appendix

Multimedia Appendix 1 Supplementary data for the compiled corpus.

Multimedia Appendix 2 Supplementary data for the developed methods.

Multimedia Appendix 3 Detailed experimental results.

Multimedia Appendix 4 Supplementary data for the discussion.

## Abbreviations

AHV: attention-head view
BERT: Bidirectional Encoder Representations from Transformers
CH-BERT: BERT pretrained on simplified and traditional Chinese corpora
CMI: code-mixing index
CMIC: code-mixing index for corpus
DBA: dictionary-based approach
EHR: electronic health record
EN-BERT: BERT pretrained on English corpora
HIPAA: Health Insurance Portability and Accountability Act
IV: in-vocabulary
LLM: large language model
M-BERT: BERT pretrained on Wikipedia corpora from 104 languages
MIMIC: Medical Information Mart for Intensive Care
NLP: natural language processing
OOV: out-of-vocabulary
PHI: protected health information
PLM: pretrained language model
TM-BERT: M-BERT fine-tuned on the translated deidentification corpus
t-SNE: t-Distributed Stochastic Neighbor Embedding
