# Supplementary material for: Unlocking the Secrets Behind Advanced Artificial Intelligence Language Models in Deidentifying Chinese-English Mixed Clinical Text: Development and Validation Study
Source: J Med Internet Res. 2024 Jan 25;26:e48443. doi: 10.2196/48443 (PMC10853853; doi:10.2196/48443)
Supplement: Multimedia Appendix 1 [file jmir_v26i1e48443_app1.docx]

Multimedia Appendix 1. Supplementary data for the compiled corpus.

The examples shown in Table S1 illustrate more the code switching and code-mixing (CM) problems occurred when we process the summaries.

**Table S1.** Example code-switched or code-mixed narratives in discharge summaries. The PHIs required to be identified in the “Example Sentence” column are shown in bold and italic, the column to the right contains the corresponding PHI type definitions.

| Example Sentences | PHI Types |
| --- | --- |
| 前夫 **mk1300309** married > **mk136** 從婆家搬出來->**mk139** 離婚 | Date |
| Persecutory delusion was reported after several workers did enter her home for help on ***2909/8/23****.* 案母找了一些鄰里間遊手好閒的年輕人來家裡幫忙搬家… | Date |
| In ***2014/06/23*** afternoon, she felt that ***菲律賓***女人又對我大聲. | Date, Nationality |
| Due to her disturbing behavior (打電話到對方***公司***吵架), her husband decided to work at ***bus company***. | General Business |
| Occupational History: ***師大***畢業後曾擔任高中***英文老師***，***30***歲之後與男友一起作生意 | School, Profession, Age |
| She remained communicating with us as 本靈 and said that she lied to us on the day of admission, 我其實不想讓***雯嫺***痛苦 | Patient |
| He went to ***高雄*** for ***直銷*** in ***2714/03*** and went to ***Malaysia*** | City, Profession, Date, Country |
| Operation Date: ***2910/11/14***手術醫師: ***林晉純*** | Date, Doctor |
| Her started to became depressed after 去***南部***掃先生家的墓. | Region |
| 照會日期:***2913/05/12*** 照會科別:***內科部*** | Date, Department |
| 病患姓名:***龍千瑜*** 病歷號碼:***420398455*** 病床號碼:***03e21sk2*** | Patient, Medical Record, ID Number |

Discharge summaries were pre-processed with the following steps: 1) the unstructured text was extracted from the textual summary and the original line breaks were retained; 2) sentences were then split by the line breaks and further segmented and tokenized by the clinical NLP tool we developed; 3) the Chinese text was further tokenized as individual characters. Table S2 shows the preprocessed results for the sample sentences in Table S1, in which tokens are separated by space.

| **Table S2.** Preprocessed results for the example sentences shown in Table S1.   \| Pre-processed Sentences \| \| --- \| \| 前 夫 mk1300309 married > mk136 從 婆 家 搬 出 來 -> mk139 離 婚 \| \| persecutory delusion was reported after several workers did enter her home for help on 2909 / 8 / 23 *.* 案 母 找了 一 些 鄰 里 間 遊 手 好 閒 的 年 輕 人 來 家 裡 幫 忙 搬 家 … \| \| in 2014 / 06 / 23 afternoon , she felt that 菲 律 賓 女 人 又 對 我 大 聲 . \| \| due to her disturbing behavior ( 打 電 話 到 對 方 公 司 吵 架 ) , her husband decided to work at bus company. \| \| occupational history : 師 大 畢 業 後 曾 擔 任 高 中 英 文 老 師 , 30 歲 之 後 與 男 友 一 起 作 生 意 \| \| she remained communicating with us as 本 靈 and said that she lied to us on the day of admission , 我 其 實 不 想 讓 雯 嫺 痛 苦 \| \| he went to 高 雄 for 直 銷 in 2714 / 03 and went to malaysia. \| \| operation date : 2910 / 11 / 14 手 術 醫 師 : 林 晉 純 \| \| her started to became depressed after 去 南 部 掃 先 生 家 的 墓 . \| \| 照 會 日 期 : 2913 / 05 / 12 照 會 科 別 : 內 科 部 \| \| 病 患 姓 名 : 龍 千 瑜 病 歷 號 碼 : 420398455 病 床 號 碼 : 03e21sk2 \| |
| --- | --- | --- | --- | --- | --- | --- | --- | --- | --- | --- | --- | --- |
