# Supplementary material for: Unlocking the Secrets Behind Advanced Artificial Intelligence Language Models in Deidentifying Chinese-English Mixed Clinical Text: Development and Validation Study
Source: J Med Internet Res. 2024 Jan 25;26:e48443. doi: 10.2196/48443 (PMC10853853; doi:10.2196/48443)
Supplement: Multimedia Appendix 2 [file jmir_v26i1e48443_app2.docx]

Multimedia Appendix 2. Supplementary data for the developed methods.

The dictionary-based approach (DBA) is one of the popular methods for de-identification which relies on pre-defined dictionaries and a lookup method. In our implementation, we compiled a dictionary consisting of tokens for all PHI types collected from the training set (*e.g.*, 林/B-Doctor, 晉/I-Doctor and 純/L-Doctor shown in Table 1). The most frequent tag associated with each token estimated on the training set is set as the token’s tag. DBA scanned the discharge summary sentence-by-sentence, divided them into individual tokens separated by whitespace, and then assigned a PHI type to each token by dictionary look-ups. If the token does not appear in the training data or there is a tie in the tag frequency, the tag “O” is outputted.

The pre-trained BERT-family models including the uncased English BERT (EN-BERT) (Devlin et al., 2019), Chinese BERT (CH-BERT) and multilingual BERT (M-BERT) were used in this study. In our implementation, an additional fully connected layer was added that takes the token embeddings from the top layer of the underling BERT-based model as input. This layer is employed to predict the probabilities of the tags for each token. When the WordPiece tokenizer splits a token into multiple pieces, we followed Virtanen et al. (2019)’s approach which takes the representation of the first piece to represent the token. The number of nodes in the fully connected layer is 81, corresponding to 80 nodes for the combination of BILU tags for the 20 fine-grained PHI types plus an additional O tag. The developed models were fine-tuned for the recognition task by minimizing the cross-entropy loss on the training set using the AdamW optimizer. For all the developed BERT-based models, the training iteration was set as 20, the batch size was set as 16, the learning rate was set as $3\times{10}^{-5}$. The training process was based on the gradual warmup method, a method that ramps up the learning rate from a small to a large value, allowing healthy convergence at the start of training. In addition to fine-tuning the above BERT-based models on the original CM corpus, we translated the sentences in our English-Chinese corpus into either English or Chinese and then fine-tuned the M-BERT model on the translated corpus. In the prediction phase, we employ identical procedures to transform the input sentences into monolingual sentences based on the language that dominates, and subsequently utilize the fine-tuned trans-M-BERT (TM-BERT) to detect PHIs.
