# Supplementary material for: Unlocking the Secrets Behind Advanced Artificial Intelligence Language Models in Deidentifying Chinese-English Mixed Clinical Text: Development and Validation Study
Source: J Med Internet Res. 2024 Jan 25;26:e48443. doi: 10.2196/48443 (PMC10853853; doi:10.2196/48443)
Supplement: Multimedia Appendix 3 [file jmir_v26i1e48443_app3.docx]

Multimedia Appendix 3. Detailed experimental results.

Figure S1 demonstrates that the numbers of PHIs in the training and test sets have similar distributions.


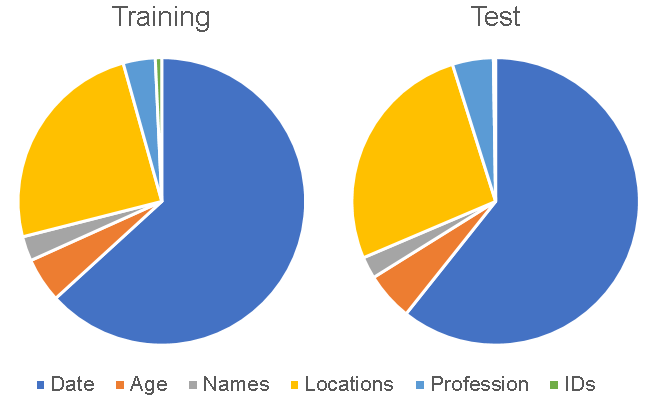


**Figure S1.** The distribution of the coarse-grained PHI types.

Table S1 shows the AvgCMI_English_, AvgCMI_Chinese_, and the code mixing index for corpus (CMIC) estimated for the training set, test set, and entire corpus. AvgCMI*_L_* is defined as

$\mathrm{AvgCMI}_{L}=\frac{\sum_{s\in K} {CMI}_{s}}{\left| K \right|}$ (C.1)

where *L* is either the English or Chinese, and *K* indicates the set of *L*-dominated sentences in our corpus. Notice that the calculation of AvgCMI*_L_* includes the sentences with the code mixing index (CMI defined in Eq. B.1) with the value of zero (monolingual sentences). CMIC is defined as follows.

$\mathrm{CMIC}=\sum_{K} \mathrm{AvgCMI}_{L}$ (C.2)

The prevalence of English-dominated sentences (220,348) compared to Chinese-dominated sentences (53,972) highlights the writing convention in Taiwan’s medical records; the majority of records being written in English. The phenomenon that English-only sentences occupies 91.5% English-dominated sentences, whereas the Chinese-only sentence occupies 34.3% Chinese-dominated sentences further reveals another interesting writing convention that CM appears more frequently in the Chinses-dominated sentences. This pattern is also reflected in the significant difference between AvgCMI_English_ and AvgCMI_Chinese_; Chinese tokens occur much less frequent in English-dominated sentences than English tokens appeared in Chinese-dominated sentences. The CMIC of our corpus is 22.21, significantly higher than the CMIC of English-Bangla (~5.15) and Dutch-Turkish (~4.13) data sets from Gambäck and Das (2014).

| **Table S1.** CM Level of the developed dataset.   \|  \| Training Set \| Test Set \| Entire Corpus \| \| --- \| --- \| --- \| --- \| \| AvgCMI_English_ \| 3.25 \| 3.18 \| 3.22 \| \| AvgCMI_Chinese_ \| 19.04 \| 18.95 \| 18.99 \| \| CMIC \| 22.29 \| 22.13 \| 22.21 \| \| Number of English-dominated sentences \| 182,649 \| 37,699 \| 220,348 \| \| Number of English-only sentences \| 167,005 \| 34,505 \| 201,510 \| \| Number of Chinese-dominated sentences \| 44,860 \| 9,112 \| 53,972 \| \| Number of Chinese-only sentences \| 15,418 \| 3,077 \| 18,495 \| |
| --- | --- | --- | --- | --- | --- | --- | --- | --- | --- | --- | --- | --- | --- | --- | --- | --- | --- | --- | --- | --- | --- | --- | --- | --- | --- | --- | --- | --- | --- | --- | --- | --- |

In the following experiments for the evaluation of the developed models, , the performance was measured by standard metrics used in previous de-identification tasks (Stubbs et al., 2015b) including recall (R), precision (P).

$\mathrm{Recall}_{t}=\frac{{TP}_{t}}{{TP}_{t}+{FN}_{t}}$ (C.1)

$\mathrm{Precision}_{t}=\frac{{TP}_{t}}{{TP}_{t}+{FP}_{t}}$ (C.2)

where ${TP}_{t}$ indicates the position and the type of a recognized PHI exactly matches the gold standard with the type *t*. ${FP}_{t}$ designates that the position or the type *t* of a recognized PHI don’t match the gold standard, while ${FN}_{t}$ represents that the gold standard PHI type *t* cannot match any predicted results. Moreover, the overall performance is accessed using the micro-F_1_-measure (mF), a weighted harmonic mean of P and R defined as follows.

$\text{Micro}\text{F}_{\text{1}}\text{measure}=\frac{2\times Precision\times Recall}{Precision+Recall}$ (C.3)

Eq. (C.3) is utilized to compute the overall mF by calculating P and R based on all PHIs present in the entire data set, irrespective of their types. mF gives equal importance to each test case, implying that the performance of the majority PHI types has higher contribution to the score than the minority types. We further report macro-F_1_-measure (MF) as the unweighted mean of the F-scores calculated per PHI types, namely, the performance of each type contributes equally to the score as defined in Eq. (C.4).

$\text{Macro}\text{F}_{\text{1}}\text{measure}=\frac{\sum_{i=0}^{n} \text{Micro}\text{F}_{\text{1}}\text{measure}}{n}$ (C.4)

All the experiments were conducted on a Windows 10 computer with Intel I9-9900K CPU, 64 GB RAM, and a Nvidia graphic card of GeForce RTX 3090 GPU with 24 GB GPU RAM. The software development was based on Python version 3.9.12.

Tables S2 and S3 show the performance comparison between EN-BERT, CH-BERT, and M-BERT in sentences classified as English-only, Chinese-only, and Chinese-English mixed in terms of P and R, respectively. According to the mFs reported in Tables S2 and S3 and the Table 2 of the original paper, M-BERT outperforms all others in the three sentence categories, while CH-BERT achieved the best MF in the Chinese-only and CM sentences. Regarding English-only sentences, EN-BERT exhibits comparable performance in both mF and MF of M-BERT and it even outperform CH-BERT. For Chinese-only sentences, it is not surprising that the performance of EN-BERT is inferior to that of CH-BERT and M-BERT by 0.43 and 0.32 in terms of MF, respectively. The differences of mF between EN-BERT and CH-BERT/M-BERT for Chinese-English mixed sentences are 0.14 and 0.16, which are notably larger than those observed in English-only and Chinese-only sentences. Moreover, these findings indicate that CM sentences pose greater difficulties for EN-BERT.

Furthermore, we observe that the disparity between MF and mF of EN-BERT is more significant in Chinese-only and CM sentences compared to that of CH-BERT and M-BERT. In particular, for the Chinese-only sentence, although the mF of EN-BERT is comparable to that of the other models, the MF of EN-BERT is significant lower indicating that the EN-BERT encounters more challenges in recognizing certain PHI types in both sentence categories. In particular, we observe that EN-BERT underperformed CH-BERT and M-BERT on PHI types, which have a limited number of training instances. Specifically, for types such as “Patient”, “Person”, “Nationality”, “Country”, “City”, “Hospital”, “School”, “Generic Location”, and “Profession”, the F-scores of EN-BERT in Chinese-only sentences are significantly lower than those of CH-BERT and M-BERT by at least 0.13.

**Table S2.** A comparison of the precision between EN-BERT, CH-BERT, and M-BERT in sentences classified as English-only, Chinese-only, and Chinese-English mixed. PHI types in bold face represent coarse-grained PHIs

| Types  PHI | English-only | | | | Chinese-only | | | | Chinese-English Mixed | | | |
| --- | --- | --- | --- | --- | --- | --- | --- | --- | --- | --- | --- | --- |
|  | EN-BERT | CH-BERT | M-BERT | Num. of PHI | EN-BERT | CH-BERT | M-BERT | Num. of PHI | EN-BERT | CH-BERT | M-BERT | Num. of PHI |
| Date | 0.95 | 0.93 | 0.95 | 2,346 | 0.98 | 0.97 | 0.99 | 458 | 0.93 | 0.93 | 0.93 | 919 |
| Age | 0.95 | 0.93 | 0.94 | 533 | 0.57 | 0.83 | 0.67 | 5 | 0.97 | 0.97 | 0.95 | 128 |
| Names | 0.21 | 0.21 | 0.21 | 18 | 0.40 | 0.61 | 0.60 | 44 | 0.62 | 0.84 | 0.80 | 240 |
| Patient | 0.00 | 0.00 | 0.00 | 9 | 0.62 | 1.00 | 1.00 | 12 | 0.56 | 1.00 | 0.88 | 11 |
| Person | 0.00 | 0.00 | 0.00 | 3 | 0.00 | 0.00 | 0.00 | 1 | 0.36 | 0.57 | 0.54 | 32 |
| Doctor | 0.62 | 0.62 | 0.62 | 6 | 0.59 | 0.83 | 0.79 | 31 | 0.94 | 0.96 | 0.98 | 197 |
| Locations | 0.70 | 0.68 | 0.83 | 1,014 | 0.37 | 0.92 | 0.84 | 1,108 | 0.38 | 0.67 | 0.65 | 1,132 |
| Named Location | 0.57 | 0.33 | 0.83 | 10 | 0.00 | 0.50 | 0.50 | 3 | 0.33 | 0.60 | 0.49 | 69 |
| Nationality | 1.00 | 0.31 | 1.00 | 7 | 0.00 | 1.00 | 1.00 | 1 | 0.00 | 0.75 | 0.82 | 10 |
| Region | 0.67 | 0.71 | 0.56 | 11 | N/A | N/A | N/A | N/A | 0.00 | 0.00 | 0.00 | 1 |
| Country | 0.84 | 0.87 | 0.93 | 52 | 0.33 | 1.00 | 1.00 | 5 | 0.67 | 1.00 | 0.90 | 44 |
| City | 0.74 | 0.72 | 0.73 | 39 | 0.33 | 1.00 | 0.86 | 6 | 0.53 | 0.93 | 0.90 | 101 |
| Hospital | 0.94 | 0.93 | 0.93 | 266 | 0.17 | 0.71 | 0.77 | 11 | 0.79 | 0.91 | 0.92 | 393 |
| Department | 0.86 | 0.86 | 0.87 | 391 | 0.92 | 0.95 | 0.94 | 542 | 0.69 | 0.73 | 0.70 | 111 |
| Room | 0.77 | 0.73 | 0.77 | 182 | 0.98 | 1.00 | 0.98 | 267 | 0.54 | 0.67 | 0.68 | 53 |
| Number | N/A | N/A | N/A | N/A | 0.99 | 1.00 | 0.99 | 256 | 0.00 | 0.00 | 0.00 | 1 |
| School | 0.62 | 0.50 | 0.83 | 6 | 0.00 | 1.00 | 0.50 | 2 | 0.64 | 0.93 | 0.88 | 159 |
| Generic Location | 0.73 | 0.54 | 0.70 | 45 | 0.00 | 1.00 | 0.90 | 15 | 0.32 | 0.76 | 0.72 | 173 |
| Market | 0.00 | 1.00 | 1.00 | 3 | N/A | N/A | N/A | N/A | 0.00 | 0.77 | 0.75 | 17 |
| Profession | 0.77 | 0.62 | 0.82 | 107 | 0.14 | 0.58 | 0.50 | 9 | 0.39 | 0.66 | 0.75 | 449 |
| IDs | N/A | N/A | N/A | N/A | 0.83 | 0.83 | 0.85 | 26 | 0.50 | 0.84 | 0.75 | 5 |
| ID Number | N/A | N/A | N/A | N/A | 0.65 | 0.65 | 0.69 | 11 | 0.25 | 0.67 | 0.50 | 2 |
| Medical Record | N/A | N/A | N/A | N/A | 1.00 | 1.00 | 1.00 | 14 | 0.75 | 1.00 | 1.00 | 3 |
| mF | 0.92 | 0.89 | 0.92 | 4,018 | 0.93 | 0.96 | 0.96 | 1,650 | 0.73 | 0.84 | 0.87 | 2,873 |
| MF | 0.65 | 0.62 | 0.73 |  | 0.46 | 0.83 | 0.78 |  | 0.48 | 0.74 | 0.72 |  |

In contrast, both CH-BERT and M-BERT exhibit less effect of the CM issue in their recognition of PHIs in CM sentences, as evidenced by their acceptable mFs. Overall, the mFs of the M-BERT are consistently better than that of the other models across all of the three categories. However, it is noteworthy that CH-BERT display slightly better MF compared to M-BERT in Chinese-only and CM sentences. On the other hand, all BERT-based models show greater disparity in English-only sentences mainly due to their failure in recognizing PHIs in the “Name” type. Finally, the F-scores of the three models for PHIs of “Date” and “Age” in CM sentences are consistently high, with scores above 0.93. This indicates that mixing Chinese and English in the same sentence has little effect on the recognition of numeric PHI types.

**Table S3.** A comparison of the recall between EN-BERT, CH-BERT, and M-BERT in sentences classified as English-only, Chinese-only, and Chinese-English mixed. PHI types in bold face represent coarse-grained PHIs Recall

| Types  PHI | English-only | | | | Chinese-only | | | | Chinese-English Mixed | | | |
| --- | --- | --- | --- | --- | --- | --- | --- | --- | --- | --- | --- | --- |
|  | EN-BERT | CH-BERT | M-BERT | Num. of PHI | EN-BERT | CH-BERT | M-BERT | Num. of PHI | EN-BERT | CH-BERT | M-BERT | Num. of PHI |
| Date | 0.96 | 0.95 | 0.96 | 2,346 | 0.98 | 0.98 | 0.99 | 458 | 0.93 | 0.93 | 0.94 | 919 |
| Age | 0.98 | 0.99 | 0.99 | 533 | 0.80 | 1.00 | 0.80 | 5 | 0.92 | 0.97 | 0.96 | 128 |
| Names | 0.28 | 0.28 | 0.28 | 18 | 0.36 | 0.49 | 0.49 | 44 | 0.51 | 0.78 | 0.77 | 240 |
| Patient | 0.00 | 0.00 | 0.00 | 9 | 0.67 | 1.00 | 1.00 | 12 | 0.45 | 0.64 | 0.88 | 11 |
| Person | 0.00 | 0.00 | 0.00 | 3 | 0.00 | 0.00 | 0.00 | 1 | 0.12 | 0.72 | 0.44 | 32 |
| Doctor | 0.83 | 0.83 | 0.83 | 6 | 0.42 | 0.48 | 0.48 | 31 | 0.95 | 0.99 | 0.99 | 197 |
| Locations | 0.65 | 0.59 | 0.71 | 1,014 | 0.36 | 0.85 | 0.83 | 1,108 | 0.37 | 0.66 | 0.67 | 1,132 |
| Named Location | 0.40 | 0.20 | 0.50 | 10 | 0.00 | 0.33 | 0.33 | 3 | 0.25 | 0.48 | 0.55 | 69 |
| Nationality | 0.50 | 0.50 | 0.50 | 7 | 0.00 | 1.00 | 1.00 | 1 | 0.00 | 1.00 | 0.75 | 10 |
| Region | 0.36 | 0.45 | 0.45 | 11 | N/A | N/A | N/A | N/A | 0.00 | 0.00 | 0.00 | 1 |
| Country | 0.91 | 0.84 | 0.98 | 52 | 0.20 | 1.00 | 1.00 | 5 | 0.60 | 0.88 | 0.86 | 44 |
| City | 0.74 | 0.72 | 0.82 | 39 | 0.17 | 1.00 | 1.00 | 6 | 0.54 | 0.78 | 0.75 | 101 |
| Hospital | 0.93 | 0.92 | 0.93 | 266 | 0.27 | 0.91 | 0.91 | 11 | 0.80 | 0.94 | 0.95 | 393 |
| Department | 0.78 | 0.77 | 0.78 | 391 | 0.98 | 0.98 | 0.99 | 542 | 0.68 | 0.80 | 0.86 | 111 |
| Room | 0.94 | 0.95 | 0.95 | 182 | 0.96 | 0.97 | 0.96 | 267 | 0.64 | 0.89 | 0.85 | 53 |
| Number | N/A | N/A | N/A | N/A | 1.00 | 1.00 | 1.00 | 256 | 0.00 | 0.00 | 0.00 | 1 |
| School | 0.83 | 0.17 | 0.83 | 6 | 0.00 | 0.50 | 0.50 | 2 | 0.66 | 0.91 | 0.92 | 159 |
| Generic Location | 0.71 | 0.64 | 0.71 | 45 | 0.00 | 0.80 | 0.60 | 15 | 0.24 | 0.71 | 0.80 | 173 |
| Market | 0.00 | 0.33 | 0.33 | 3 | N/A | N/A | N/A | N/A | 0.00 | 0.59 | 0.71 | 17 |
| Profession | 0.79 | 0.84 | 0.81 | 107 | 0.22 | 0.78 | 0.78 | 9 | 0.34 | 0.75 | 0.76 | 449 |
| IDs | N/A | N/A | N/A | N/A | 1.00 | 1.00 | 1.00 | 26 | 0.75 | 1.00 | 0.75 | 5 |
| ID Number | N/A | N/A | N/A | N/A | 1.00 | 1.00 | 1.00 | 11 | 0.50 | 1.00 | 0.50 | 2 |
| Medical Record | N/A | N/A | N/A | N/A | 1.00 | 1.00 | 1.00 | 14 | 1.00 | 1.00 | 1.00 | 3 |
| mF | 0.92 | 0.92 | 0.93 | 4,018 | 0.94 | 0.97 | 0.97 | 1,650 | 0.69 | 0.87 | 0.88 | 2,873 |
| MF | 0.63 | 0.59 | 0.67 |  | 0.48 | 0.82 | 0.80 |  | 0.48 | 0.75 | 0.72 |  |
