# Supplementary material for: Unlocking the Secrets Behind Advanced Artificial Intelligence Language Models in Deidentifying Chinese-English Mixed Clinical Text: Development and Validation Study
Source: J Med Internet Res. 2024 Jan 25;26:e48443. doi: 10.2196/48443 (PMC10853853; doi:10.2196/48443)
Supplement: Multimedia Appendix 4 [file jmir_v26i1e48443_app4.docx]

Multimedia Appendix 4. Supplementary data for the discussion.

In this study, the ambiguity issue could arise within or between coarse-grained PHI types. When a fine-grained PHI and its actual counterpart are misclassified, they may belong to the same coarse-grained PHI or different ones. It is more acceptable to misclassify them under the same coarse-grained PHI since fine-grained PHIs share similar semantic characteristics. Herein, we investigate the issue of ambiguity by mapping the predicted fine-grained PHI types of M-BERT to their coarse-grained PHI type for the estimation of mapped micro-F-scores and compared them to the original scores of the coarse-grained PHI types. The mapping process considers an incorrect recognition of a fine-grained PHI type as a true positive (TP) if it belongs to the same coarse-grained PHI type as the corresponding gold annotation. Table S1 presents the results. Figure S1 further displays the confusion matrix generated for the M-BERT.

**Table S1.** Comparison of F-scores of coarse-grained PHIs using unweighted mean (macro-F) and the mapping of the fine-grained PHIs (micro-F-scores) of M-BERT on the test set.

| Coarse-grained PHI | P | R | Macro-F | P | R | Micro-F |
| --- | --- | --- | --- | --- | --- | --- |
| Names | 0.87 | 0.68 | 0.75 | 0.99 | 0.95 | 0.97 |
| Locations | 0.78 | 0.74 | 0.75 | 0.99 | 0.99 | 0.99 |
| IDs | 0.81 | 1.00 | 0.88 | 0.97 | 1.00 | 0.98 |


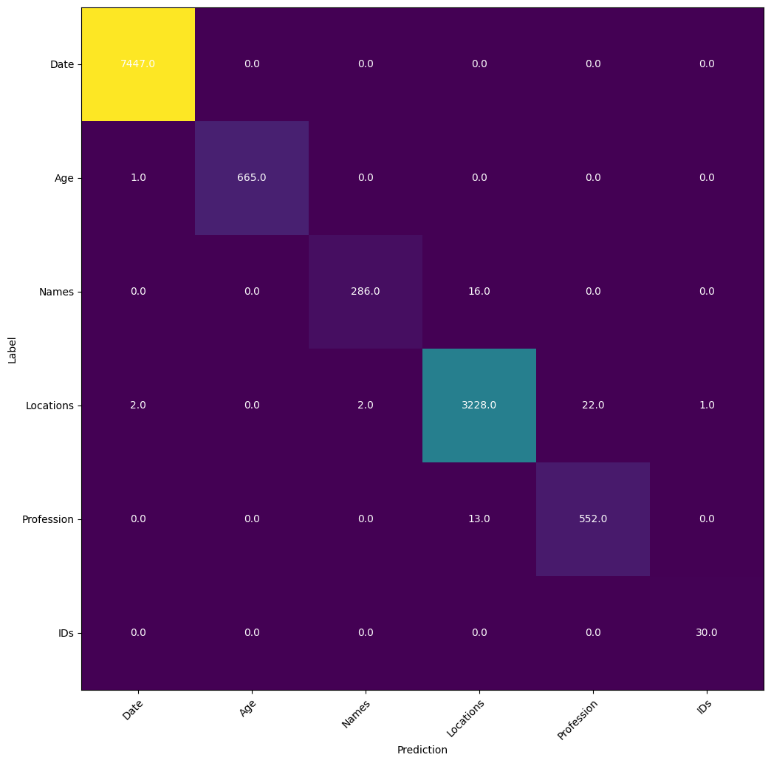


**Figure S1.** The confusion matrix for the mapped results of M-BERT.

It can be seen from Table S1 that the mapped F-scores for “Names” and “Locations” are 29% and 32% higher than the unmapped F-scores. In contrast, the coarse-grained PHI of “IDs” has merely a 11% difference. Based on the results, it appears that intra-type ambiguities are more prevalent than inter-type ambiguities in “Names” and “Locations”. To illustrate the ambiguity appeared in the Locations PHI, consider the following two sentences: “He was transferred to **八里** for further treatment ...” and “上**台大**夜外文，念了一年又無法繼續.” In the first sentence, “八里 (Bali)” should be classified as a hospital (which all developed BERT-based models can successfully do), while in the second sentence, “台大” should be recognized as the National Taiwan University instead of National Taiwan University Hospital (NTUH) (only CH-BERT can classify the type accurately). Figure S2 displays the representations generated by M-BERT for the PHI types of “Nationality”, “Country”, “Hospital”, “School”, and “Region” through t-SNE. To serve as a comparison, we include a semantically distinct PHI, “ID”, in the plot. The visualization provides insight into the level of ambiguity among PHI types. The clusters of “Nationality”, “Country” and “Region” are intertwined, while the cluster of “ID” is notably separated from the others. We believe that the high ambiguity may be owing to the limited number of training instances. For example, the number of training instances is imbalanced among PHIs, with “Country” having 633 training instances, which is considerably larger than “Nationality” and “Region”, which have only 59 and 28 training instances, respectively. As a result, all BERT-based models yield better F-scores for “Country” than for “Nationality” and “Region.”

| 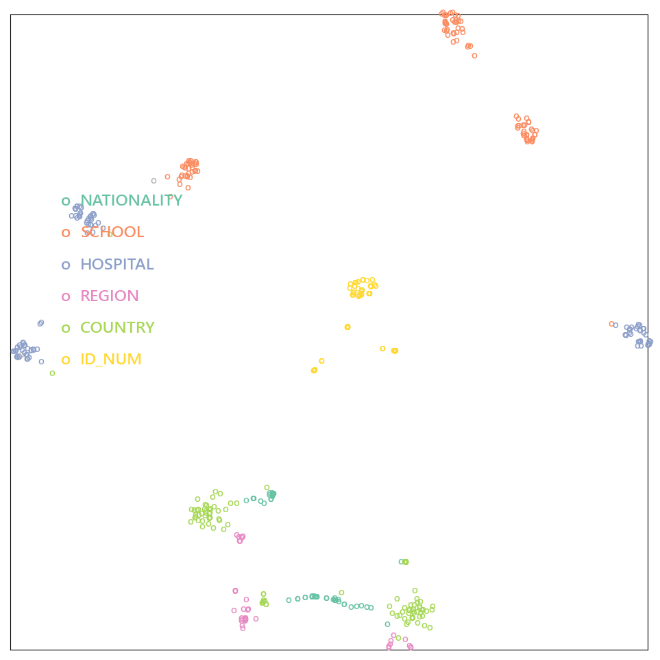  **Figure S2.** t-SNE visualization of the five fine-grained “Locations” and the “ID” PHI types for M-BERT. |
| --- |

Name regularity refers to the consistency and predictability of the structure and pronunciation of a named entity types within a particular language. For example, person names typically adhere to the “FirstName LastName” or “LastName FirstName” formats, while medical record identifiers mostly begin with indicator phrases such as “病歷號碼:” or “病理號:”. A high mention coverage denotes that a large proportion of mentions in the test set have been previously observed in the training set. Research studies conducted by Lin et al. (2020) and Kim and Kang (2021) have demonstrated that that state-of-the-art neural network models can easily exploit the strong name regularity and high mention coverage to achieve superior performance. However, these models may exhibit suboptimal results when the regularity is weak, such as in the case of unseen mentions or out-of-vocabulary (OOV) words, or may solely memorize popular mentions instead of learning generalization knowledge.

**Figure S3.** F-scores of coarse-grained PHIs for tokens belonging to IV and OOV in the test set.

The inadequate performance by DBA indicates that the mention coverage in our corpus is restricted. The OOV rates estimated on the test set are 0.356, 0.953, 0.686, 0.876, 0.696, and 0.29 for the categories of "Date", "Age", "Names", "Locations", "Profession", and "IDs", respectively. Here an OOV is defined as the mention that comprises of at least one word that is not present in the vocabulary compiled from the training dataset. In order to address the problem of OOV, the model should learn the context pattern from an adequate training data that can provide sufficient context diversity. To analyze the effect of coverage of mention, Figures S3-S5 respectively shows the F-, P- and R-scores of coarse-grained PHIs for tokens belonging to in-vocabulary (IV) and OOV using EN-BERT, M-BERT and TM-BERT.

**Figure S4.** Precision of the coarse-grained PHIs for tokens belonging to IV and OOV in the test set.

**Figure S5.** Recall of coarse-grained PHIs for tokens belonging to IV and OOV in the test set.

Finally, the estimated frequencies of unknown words for EN-BERT, CH-BERT, and M-BERT can be found in Table S2.

**Table S2.** The unknown word frequencies estimated on the training and validation sets for different BERT-based pre-trained models.

|  | Training Set | | | Validation Set | | |
| --- | --- | --- | --- | --- | --- | --- |
| Coarse-grained PHI | EN-BERT | CH-BERT | M-BERT | EN-BERT | CH-BERT | M-BERT |
| English | 0% | 0% | 0% | 0% | 0% | 0% |
| Chinese | 27.8% | 0.45% | 0.2% | 29.3% | 0.12% | 0.27% |
| Special characters | 0.29% | 0.52% | 0.25% | 0.25% | 0.26% | 0.26% |
